# Survey on Pain Detection Using Machine Learning Models: Narrative Review

**DOI:** 10.2196/53026

**Published:** 2025-02-24

**Authors:** Ruijie Fang, Elahe Hosseini, Ruoyu Zhang, Chongzhou Fang, Setareh Rafatirad, Houman Homayoun

**Affiliations:** 1 Department of Electrical and Computer Engineering University of California Davis, CA United States; 2 Department of Computer Science University of California Davis, CA United States

**Keywords:** pain, pain assessment, machine learning, survey, mobile phone

## Abstract

**Background:**

Pain, a leading reason people seek medical care, has become a social issue. Automated pain assessment has seen notable advancements over recent decades, addressing a critical need in both clinical and everyday settings.

**Objective:**

The objective of this survey was to provide a comprehensive overview of pain and its mechanisms, to explore existing research on automated pain recognition modalities, and to identify key challenges and future directions in this field.

**Methods:**

A literature review was conducted, analyzing studies focused on various modalities for automated pain recognition. The modalities reviewed include facial expressions, physiological signals, audio cues, and pupil dilation, with a focus on their efficacy and application in pain assessment.

**Results:**

The survey found that each modality offers unique contributions to automated pain recognition, with facial expressions and physiological signals showing particular promise. However, the reliability and accuracy of these modalities vary, often depending on factors such as individual variability and environmental conditions.

**Conclusions:**

While automated pain recognition has progressed considerably, challenges remain in achieving consistent accuracy across diverse populations and contexts. Future research directions are suggested to address these challenges, enhancing the reliability and applicability of automated pain assessment in clinical practice.

## Introduction

Pain is “an unpleasant sensory and emotional experience associated with actual or potential tissue damage, or described in terms of such damage,” according to the International Association for the Study of Pain [1]. However, the discussion on the most precise definition of pain is still ongoing, and the advances in the understanding of pain instantiate the biopsychosocial perspective on pain to capture evidence-based understanding and the evolution of pain [2]. On the basis of the pain origin, it is categorized as nociceptive (due to stimulation of sensory nerve fibers), neuropathic (due to impaired somatosensory nervous system), or psychogenic pain (caused, increased, or prolonged by mental, emotional, or behavioral factors). On the basis of the time duration of the pain, it may be categorized as acute (short duration) or chronic (long duration, may last >3 months).

Approximately 20% of adults have chronic pain in the United States, and chronic pain is the most common reason adults seek medical care. For society, chronic pain contributes to an estimated US $560 million each year in medical expenses, lost productivity, and disability caused by types of pain such as low back pain, arthritis, and joint pain [3,4]. These negative impacts make chronic pain a persistent public health concern. Inappropriate pain management can lead to very deleterious physical, psychological, social, and financial consequences for patients. Untreated pain can lead to chronic pain syndrome, which is often accompanied by decreased mobility, impaired immunity, decreased concentration, anorexia, and sleep disturbances. More importantly, the use of prescription opioids for the treatment of chronic noncancer pain is associated with a substantial risk for abuse, dependence, and overdose [5].

As the first step of pain management, pain assessment holds an essential role [6]. Unprecise pain assessment can lead to severe consequences. Undertreatment of pain not only causes psychological consequences but also physiological consequences, for example, increased blood pressure and heart rate. By contrast, overtreatment of pain may result in nausea, vomiting, or constipation immediately and drug addiction in the long term. Traditionally, pain assessment is conducted through self-reports or observational scales. Self-report refers to the conscious communication of pain-related information by the person in pain, typically using spoken or written language or gestures. Various pain rating scales have been developed to capture patients’ self-report of pain intensity. Traditional approaches used to play an important role in pain assessment, including the Verbal Rating Scale [7], the Visual Analog Scale [8], the Numerical Rating Scale [9], and the Wong-Baker FACES Scale [10].

However, such scoring methods are not feasible for certain patients, such as such as those who are unconscious. For this, different observational pain scales, such as the Behavioral Pain Scale [11], Pain Assessment in Advanced Dementia [12], or Neonatal Infant Pain Scale [13], are used in clinical settings. Most scales consider facial expressions, vocalizations, and body language, while some include vital parameters. It is difficult to assess and compare the validity of the various scales because studies differ a lot in design, methodology, participants, and conceptualization of the pain phenomenon. Pain assessment through observation is very challenging and is affected by the subjective biases and errors in beliefs of the observer [14].

**Figure 1:**
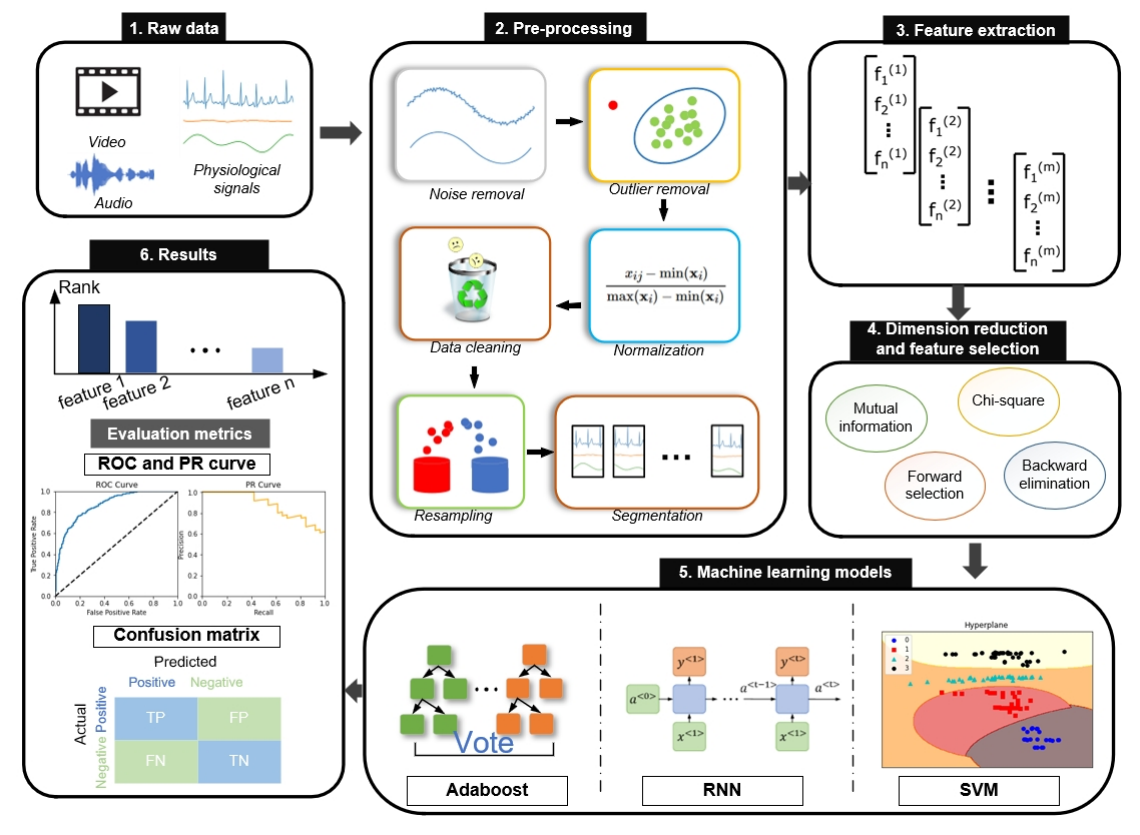
Typical pipeline of automated pain assessment. FN: false negative; FP: false positive; PR: precision-recall; RNN: recurrent neural network; ROC: receiver operating characteristic; SVM: support vector machine.; TN: true negative; TP: true positive.

To solve these challenges, it is necessary to develop an objective, accurate, continuous pain assessment method, as shown in Figure 1. In the last decades, multiple studies have been conducted to evaluate the feasibility of automated pain assessment using multimodality and machine learning (ML) techniques. This paper surveys and reviews the recent advances in the field in terms of datasets, modalities, and ML models. Finally, we present the challenges remaining in the field and propose future directions.

## Pain Mechanism

**Figure 2:**
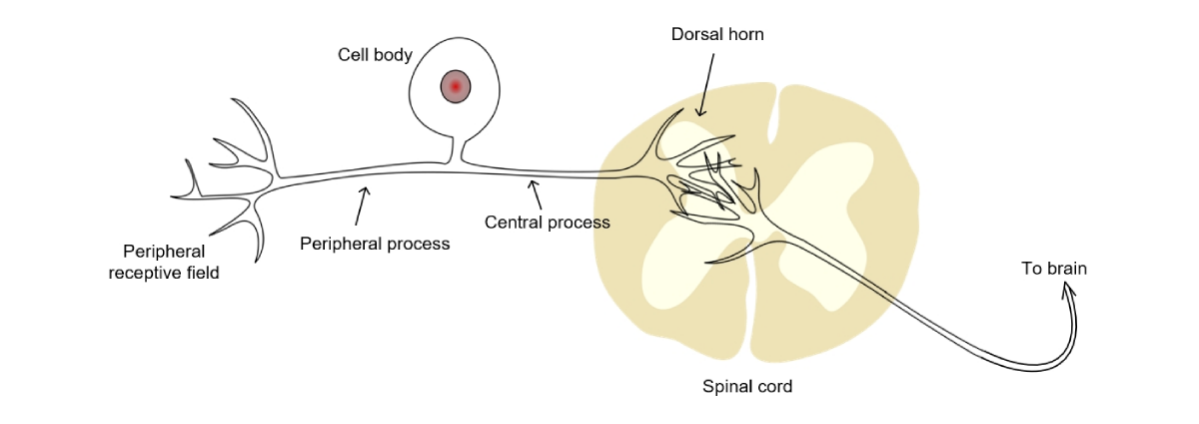
Pain mechanism.

The pain mechanism is not completely understood because of its complexity and diversity [15]. Pain, created by the brain, is a psychological state rather than a physical one [16]. Unlike pain, nociception refers to the response of the peripheral and central nervous systems to internal or external stimuli, triggered by the activation of nociceptors [17]. The noxious stimulus damages the tissue or potentially activates the nociceptors in the peripheral structure. Then, the information is transmitted to the spinal cord dorsal horn or the nucleus caudalis. From there, the information continues to the cerebral cortex via the brainstem in the brain, and the perception of pain is generated. Thus, no brain, no pain [18]. Figure 2 presents the mechanism of pain.

Usually, pain is regarded as chronic or acute according to its duration. Acute pain is a type of sudden pain. The mechanism of momentary pain is well understood [19]. The nociceptors generate the nociception, and the information is transmitted to the brain, where the perception of pain is caused. There are 2 major types of nociceptors responding to different stimuli: C-fibers, associated with unmyelinated axons, and A-delta fibers, associated with thinly myelinated axons [20]. C-fibers generate slow, diffuse pain, while A-delta fibers are related to sharp, pricking pain. Silent nociceptors typically respond to endogenous chemical mediators related to tissue injury [19].

Chronic pain, lasting >3 months, does not have a useful biological function and is challenging to treat due to its varied etiologies [21-23]. According to the *International Classification of Diseases, Eleventh Revision*, chronic pain can be categorized into musculoskeletal, neuropathic, visceral, and cancer pain [21].

Psychological distress refers to a diffuse subjective experience as an internal response to noxious stimuli. Many patients argue that psychological pain is more severe than intense physical pain [24]. Chronic pain can lead to psychological pain and depression, while depression can exacerbate chronic pain [25,26]. Psychogenic pain is physical pain caused or increased by mental and emotional factors [27]. Treatments such as transcutaneous electrical nerve stimulation or psychotherapy are often more effective for reducing psychogenic pain compared to traditional painkillers [28,29].

The body responds to pain via multiple physiological processes: the sympathetic nervous system (SNS), neuroendocrine system, immune system, as well as emotions [30]. The SNS, known for the fight or flight response, increases heart rate and blood pressure via hormones such as catecholamines, epinephrine, and norepinephrine when activated [31]. The SNS also activates sweat glands via acetylcholine, reflecting the active level of SNS through the volume of secreted sweat within a time range [32].

## Pain Datasets

Data that are representative are crucial in the creation of a pain recognition system and the demonstration of its efficacy. Crucially, the system should perform optimally within the intended medical context, a fact that must be validated through clinical studies involving patients. In the early stages of development, experimental pain research with healthy volunteers could be useful. This approach allows for strictly controlled conditions, larger participant pools, and the repeated application of pain stimuli. These data are foundational to the development of ML models for automated pain detection.

For studying pain in healthy adults, an external stimulus is needed. Common methods include heat applied via contact (eg, heated objects and electrical heaters) or radiant sources (eg, infrared light). Table 1 summarizes the publicly available datasets that were used for pain recognition research. The UNBC-McMaster Shoulder Pain Expression Archive Database [33] includes 200 video sequences that capture the facial expressions of 25 participants experiencing shoulder pain. Each video sequence includes individuals performing a series of active and passive range-of-motion tests to provoke visible responses to pain, providing a unique dataset rich in both the variety and volume of pain expressions. The dataset includes self-reported and observer assessments of pain intensity at the video level, along with Facial Action Coding System (FACS) coding at the frame level. The BioVid Heat Pain Database [34] is a collection of physiological data and videos from 90 healthy adults subjected to controlled heat stimuli. BioVid consists of several sections: A, B, and C, which focus on pain stimulation, along with sections D and E, which are dedicated to posed expressions and emotion elicitation, respectively. The MIntPAIN database [35] collected color, depth, and thermal videos from 20 healthy adults who were subjected to approximately 1600 instances of electrical pain stimuli at 4 different intensity levels. EmoPain [36], SenseEmotion [37], X-ITE Pain [38], BP4D-Spontaneous [39], and BP4D+ [40] datasets are substantially resources for pain and emotion studies. EmoPain contains video, audio, motion, and a surface electromyogram (sEMG) for lower back pain. SenseEmotion and X-ITE Pain include audio and physiological data from healthy adults subjected to experimental pain stimuli, while X-ITE provides thermal videos, body movement data, and electromyography measurements. BP4D-Spontaneous and BP4D+ offer facial video recordings from individuals undergoing the cold presser task, with BP4D+ further providing 3D and thermal videos, along with physiological signals.

**Table 1:** Pain databases.

Database			Participants		Modalities		Annotation
**Database with adults**							
	UNBC-McMaster [33]	25 adults with shoulder pain		Video of the face (RGB^a^)		FACS^b^, VAS^c^, and OPI^d^	
	BioVid [34]	87 healthy adults		Video of face (RGB), EDA^e^, electrocardiogram, and electromyography		Stimulus (calibrated per person)	
	MIntPAIN [35]	20 healthy adults		Video of face (RGB, depth, and thermal)		Stimulus (calibrated per person)	
	EmoPain [36]	22 adults with chronic back pain		Video, audio, electromyography, and motion capture		Self-report and naive OPI	
	SenseEmotion [37]	45 healthy adults		Video of face, audio, EDA, electrocardiogram, and electromyography		Stimulus (calibrated per person)	
	X-ITE [38]	134 healthy adults		Video of face, video of body, audio, EDA, electrocardiogram, and electromyography		Stimulus (calibrated per person)	
	BP4D-spontaneous [39]	41 healthy adults		Video of face (RGB and 3D)		Stimulus and FACS	
	BP4D+ [40]	140 healthy adults		Video of face (RGB, 3D, and thermal), heart rate, respiration rate, blood pressure, and EDA		Stimulus and FACS	
**Database with neonates**							
	iCOPE [41]	26 healthy neonates		204 RGB photographs of face		Category (pain, rest, cry, air puff, and friction)	
	YouTube [42]	142 infants		Video and audio		FLACC^f^	
	APN-db [43]	112 healthy neonates		Video of face (RGB)		NFLAPS^g^, NIPS^h^, and NFCS^i^	
	NPAD-ID [44]	36 healthy neonates and 9 neonates who underwent surgery		Video of face and body (RGB)		NIPS and N-PASS	
	iCOPEvid [45]	49 neonates		Video of face (grayscale)		Category (pain and no pain)	
	USF-MNPAD-I [46]	36 neonates		Video of face (RGB), audio, heart rate, blood pressure, SpO_2_^j^, deoxyhemoglobin (HbH), oxyhemoglobin (HbO_2_)		NIPS and N-PASS^k^	

^a^RGB: Red, green, blue color model.

^b^FACS: Facial Action Coding System.

^c^VAS: Visual Analog Scale.

^d^OPI: Observed Pain Intensity.

^e^EDA: electrodermal activity.

^f^FLACC: Face, Legs, Activity, Cry, Consolability Scale.

^g^NFLAPS: Neonatal Face and Limb Acute Pain Scale

^h^NIPS: Neonatal Infant Pain Scale.

^i^NFCS: Neonatal Facial Coding System.

^j^SpO_2_: saturation of peripheral oxygen.

^k^N-PASS: Neonatal Pain, Agitation and Sedation Scale.

In the field of infant pain research, the iCOPE [41], YouTube [42], APN-db [43], iCOPEvid [45], and USF-MNPAD-I [46] databases are the publicly available datasets. The iCOPE consists of 204 static photographs that capture 26 neonates during various procedures. The images provide valuable insights into the facial expressions associated with infant pain experiences. The YouTube dataset offers 142 videos accompanied by audio, showcasing the reactions of different infants undergoing immunizations. The APN-db is a dataset that includes >200 videos of infants undergoing various procedures, and it features unique annotations, such as Neonatal Face and Limb Acute Pain intensity. The USF-MNPAD-I dataset collects video, audio, and physiological data from 58 neonates during their hospitalization in the neonatal intensive care unit (ICU) and is annotated using the Neonatal Infant Pain Scale and N-PASS scales.

## Postoperative Pain

Although automated pain assessment in controlled settings is well studied, postoperative pain has not been extensively researched due to the difficulty of data collection. Postoperative pain results from tissue injury following surgery and is critical to manage, as inadequate treatment can lead to serious physiological and psychological outcomes. Postoperative pain datasets often exhibit imbalanced distributions and may contain missing labels due to variability in patient experiences and clinical settings, further complicating accurate and comprehensive pain assessment. The NPAD-IA database [44] captures video, audio, and physiological data from 40 infants undergoing procedural (heel lancing and immunization) and postoperative (gastrostomy tube) pain. Notably, it includes postoperative pain data, addressing the complexity and variability of pain levels in real-world clinical settings, thereby enhancing the ecological validity of the assessment. Salekin et al [47] present a novel fully automated deep learning framework to assess neonatal postoperative pain. It uses a bilinear convolutional neural network (B-CNN) to extract facial features and a recurrent neural network (RNN) to model the temporal patterns of postoperative pain. The study uses a dataset of >600 minutes of visual, vocal, and physiological data from neonates, demonstrating the feasibility and efficiency of combining B-CNN and RNN for continuous and accurate assessment of postoperative pain intensity in clinical settings. Salekin et al [46] introduce an automated system for assessing neonatal postoperative pain by integrating visual, vocal, and physiological data. The study also uses a B-CNN for spatial feature extraction but uses a long short-term memory (LSTM) network for capturing temporal patterns, demonstrating that the multimodal spatial-temporal approach significantly outperforms unimodal methods, achieving an area under the curve (AUC) of 0.87 and accuracy of 79%. Automated postoperative pain assessment is still in its nascent stages, primarily hindered by a lack of comprehensive datasets and consistent research efforts. The current methods, often unimodal and focused on short-term procedural pain, fail to capture the complex and prolonged nature of postoperative pain. There is a pressing need for more extensive and diverse datasets to improve the accuracy and reliability of these systems. Despite these challenges, the potential benefits of automated pain assessment are immense, offering more consistent and objective pain management that can significantly enhance patient outcomes and reduce the burden on health care providers.

## Automatic Pain Assessment

### Overview

Automated tools for pain assessment have great promise. Because pain results in different physiological and behavioral responses, signals that capture these may be used to detect the presence of pain. However, prior research work has been limited, and automated approaches have not yet become widely used in clinical practice. In this section, we briefly outline the different approaches relevant to the development of automated pain assessment methods described in the research literature. Specifically, we review their system architecture (inputs and outputs) and describe the data sources available for the research and development of ML-based automated pain assessment tools, together with an overview of system validation challenges. This section summarizes the results of the survey of automatic pain detection approaches.

### The Use of Modalities

The selection of sensors is a critical aspect of automated pain assessment, as different sensors can convey varying levels of information and have different discriminative abilities. Modalities commonly used in this field can be broadly classified into 3 categories: video, audio, and physiological signals, as shown in Table 2. Functional magnetic resonance imaging (fMRI) was found to be the most prevalent sensor in pain studies, with a prevalence score of 95.9. Electroencephalogram and electrocardiogram were also frequently used, with prevalence scores of 69.6 and 39.1, respectively. In contrast, functional near-infrared spectroscopy (fNIRS) and photoplethysmography had much lower prevalence scores of <10. Moreover, Multimedia Appendix 1 also includes information on modalities used in studies (including brain activity, cardiovascular activity, electrodermal activity (EDA), respiration activity, and pupil size). In terms of physiological signals, brain activity can be measured using electroencephalograms, fMRI, and fNIRS. Cardiovascular activity can be measured using an electrocardiogram or photoplethysmography, while EDA is often measured by skin conductance level or sEMG. To gain insight into the prevalence of each modality, we conducted a search for “Modality AND Pain AND Machine learning” (eg, “EEG AND Pain AND Machine learning”) on PubMed and Scopus, limiting the search to the period from January 1, 2010, to August 1, 2023. We then recorded the number of results and normalized them to the range of (0-100) for each database. The prevalence scores were then calculated as the average of the normalized results from PubMed and Scopus.

**Table 2:** Summary of the commonly used modalities.

Category and name			Description		Prevalence^a^		References
**Video**							
	Video analysis	Analyzes facial expressions and body movements to assess pain levels [48].		100		[33,35]	
**Audio**							
	Audio analysis	Analyzes vocal characteristics and speech patterns to assess pain [49].		48.2		[49]	
**Pupil size**							
	Pupil size measurement	Measures changes in pupil diameter as an indicator of pain [50].		12.7		[51,52]	
**Brain activity**							
	Electroencephalogram	It is a test that detects tiny electrical charges that result from the activity of brain cells [53].		69.6		[54-56]	
	Functional magnetic resonance imaging	It uses magnetic resonance imaging to measure the changes in hemodynamics caused by neuronal activity [57].		95.9		[58-60]	
	Functional near-infrared spectroscopy	It uses scattering arising from the main components of blood upon exposure to near-infrared light (600 nm to 900 nm) to measure changes in oxyhemoglobin and deoxyhemoglobin during brain activity [50].		7.9		[61,62]	
**Cardiovascular activity**							
	Electrocardiogram	It is a test that measures the electrical activity of the heartbeat [63].		39.1		[64-66]	
	Photoplethysmograph	It is an optical technique that can be used to detect blood volume changes in the microvascular bed of tissue [58].		9.4		[65,67]	
**Electrodermal activity**							
	Skin conductance level	It is the measurement of the electrical conductivity of the skin [60].		25.9		[65,66,68]	
	Surface electromyogram	It is a technique to measure muscle activity noninvasively using surface electrodes placed on the skin overlying the muscle [61].		25.6		[66,69,70]	
**Respiration**							
	Respiration	Respiration refers to a person’s breathing and the movement of air into and out of the lungs [66].		17.5		[69,71]	

^a^Prevalence is measured by the weighted search results from Scopus and PubMed, covering the period from 2010 to 2023, using the keywords “Name” AND “Pain” AND “Machine learning” as of August 1, 2023; the results are standardized on a scale of 0 to 100.

As shown in Table 2, video was found to be the most prevalent sensor in pain studies, with a prevalence score of 100. fMRI, electroencephalogram, and electrocardiogram were also frequently used, with prevalence scores of 95.9, 69.6, and 39.1, respectively. In contrast, fNIRS and photoplethysmography had much lower prevalence scores of <10.

Convenience and feasibility should also be considered when selecting sensors. For example, some sensors such as electroencephalograms and fMRI are nonwearable and can be invasive, which may limit their utility in certain settings. Moreover, complex signals require more sophisticated processing techniques and computing resources, which may not be practical in some situations, such as those involving microprocessors.

### Facial Expression

#### Overview

Facial expression during the experience of pain is not unspecific grimacing but conveys pain-specific information. Studies investigating facial expressions of pain have most often used FACS [48], the gold standard for facial expression research. FACS is a fine-grained, objective, and anatomically based coding system that differentiates between 44 facial movements known as action units (AUs). Coders are trained to apply specific operational criteria to determine the onset and offset as well as the intensity of the AUs. Using FACS, it was shown that facial expressions of pain are composed of a small subset of facial activities, namely, lowering the brows (AU4), cheek raise or lid tightening (AUs 6 and 7), nose wrinkling or raising the upper lip (AUs 9 and 10), and eye closure for >0.5 seconds (AU 43). Prkachin and Solomon [72] developed the Prkachin and Solomon Pain Intensity metric based on this observation, which is a 16-level scale based on the contribution of the individual intensity of pain-related AUs and is defined as follows:

*Pain*=*AU*4+(*AU*6,*AU*7)+(*AU*9+*AU*10)+*AU*43

**Figure 3:**
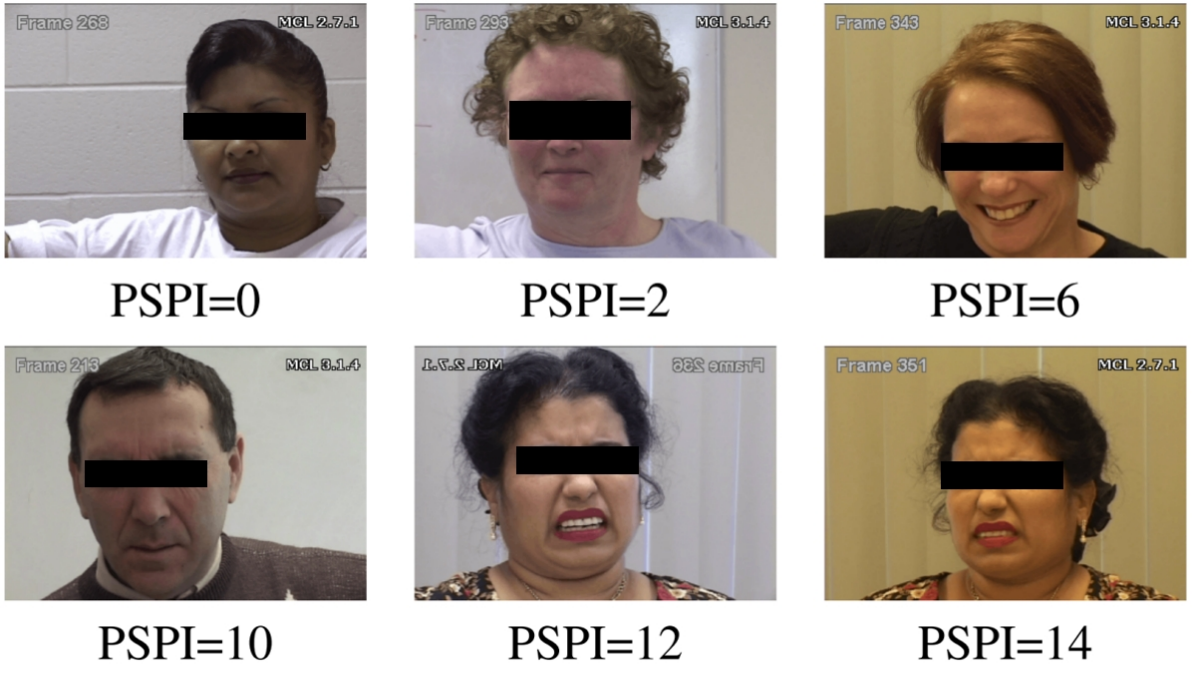
Image frame samples of the UNBC-McMaster shoulder pain database. PSPI: Prkachin and Solomon Pain Intensity.

Figure 3 shows samples of different PSPI levels from UNBC-McMaster pain dataset. The list of pain-related AUs has been further expanded in more extensive research [73] to include lip corner puller (AU12), lip stretch (AU20), lips part (AU25), jaw drop (AU26), and mouth stretch (AU27).

Facial activities during experimental and clinical pain are largely inborn but not uniform across individuals. People display different parts or combinations of facial activities. Cluster analyses identified four distinct facial activity patterns: (1) narrowed eyes with raised upper lip or nose wrinkling and furrowed brows, (2) narrowed eyes with furrowed brows, (3) narrowed eyes with mouth opening, and (4) raised eyebrows, which are less frequent and stable, often indicating novelty or surprise in response to pain. Recognizing these patterns improves pain detection more than focusing on a single expression. Thus, acknowledging variability in facial expressions can enhance pain communication.

Facial expression analysis uses spatial and spatiotemporal features. Spatial features capture static details of the face, such as the geometric and textural characteristics of the eyes, eyebrows, nose, lips, and facial contours, using techniques such as facial landmark detection, geometric feature extraction, Gabor filters, local binary patterns (LBPs), and histogram of oriented gradients (HOG). Spatiotemporal features capture dynamic changes in expressions over time using techniques such as optical flow or differences between consecutive frames. Advanced methods may involve 3D facial modeling or LSTM networks to identify temporal dependencies. Combining spatial and spatiotemporal features provides a comprehensive analysis of facial expressions.

#### Vision-Based Spatial Features

In the research conducted by Ashraf et al [74] and Lucey et al [75], features derived from the Active Appearance Model were input into support vector machine (SVM) classifiers for the purpose of frame-level pain recognition. In addition, they implemented pain detection at the sequence level by averaging the frame-level predictions. Gholami et al [76] used a Bayesian extension of SVM, known as the relevance vector machine, to differentiate between instances of pain and no pain in neonates. They also used this methodology to assess varying pain intensity levels. Meanwhile, Hammal et al [77] identified 4 levels of pain intensity through the use of log-normal filter-based features and an SVM classifier. Kaltwang et al [78] conducted a comparative study involving 3 separate methodologies. They used facial landmarks, discrete cosine transform, and LBP features to train 3 distinct relevance vector regression (RVR) models for estimating Prkachin and Solomon Pain Intensity. The best results were achieved by training an additional RVR model that consolidated the predictions from the 3 previously trained RVR models. The system [79] used a pyramid HOG for shape information and a pyramid LBP for appearance information, offering a more automated and objective approach to pain monitoring.

Pedersen [80] implementation used a 4-layer contractive autoencoder, along with SVM, which resulted in an effective pain detection system at the frame level. Egede et al [81] extracted features using both deep learning models and handcrafted methodologies. Facial landmarks, HOG, and deep vectors drawn from pretrained VGG-16 [82] and ResNet-50 [83] models were used. Rudovic et al [84] introduced a personalized federated deep learning technique for pain estimation derived from facial images. This approach involved using a compact convolutional neural network (CNN) architecture across various clients without the need to share their facial images. Contrary to the full sharing of model parameters, the personalized federated deep learning technique keeps the last layer localized. Hosseini et al [85] used a pretrained ResNet-18 model on the large emotion recognition dataset FER+ [86] and used transfer learning techniques to improve accuracy and performance. Huang et al [87] proposed a pain-awareness multistream CNN approach for feature extraction, focusing on specific regions most relevant to pain expression instead of entire face images. Semwal and Londhe [88] proposed an Ensemble of Compact CNNs using 3 compact CNNs (variants of VGG, MobileNet, and GoogleNet) and integrating their predictions using the average ensemble rule. Kharghanian et al [89,90] developed a 4-layer convolutional deep belief network, trained as convolutional restricted Boltzmann machines to extract features. Semwal et al [91] introduced a novel fusion method for pain severity assessment in unconstrained environments using a decision-level fusion of 3 distinct features: data-driven red, green, blue color model (RGB) features, entropy-based texture features, and complementary features from both RGB and texture data. Using 3 CNNs (VGG-TL, ETNet, and DSCNN) with transfer learning, entropy texture network, and dual stream CNN, the model and various data augmentation techniques avoid overfitting and improve performance. The system demonstrates a 94% *F*_1_-score on a self-generated dataset from an unconstrained hospital setting.

Alghamdi and Alaghband [92] presented a facial expressions–based automatic pain assessment system using 2 concurrent subsystems that analyze both the full face and upper half of the face through pretrained CNNs, such as VGG16, InceptionV3, ResNet50, or ResNeXt50. Dai et al [93] developed a real-time pain detection system by mixing pain and emotion datasets for optimal real-time performance and conducting a cross-corpus test. The study experiments with both AU-based and non–AU-based methods, ultimately implementing the method on a robot for frozen shoulder therapy, thus emphasizing the need for balanced and ecologically valid pain datasets and the importance of real-world application and testing. Karamitsos et al [94] use the Haarcascade frontal face detector (OpenCV) for face detection; then, faces undergo gray scaling, histogram equalization, cropping, mean filtering, and normalization. The CNN is built upon a modified VGG16 architecture, achieving an impressive 92.5% accuracy. Barua et al [95] used a shutter blinds–based model inspired by spontaneous facial expressions and patch-based learning to achieve >95% accuracy in pain detection from facial images, leveraging transfer learning for efficient deep feature extraction. The model uniquely uses horizontal dynamic-sized patches, or “shutter blinds,” to mine hidden facial signatures. Semwal et al [91] assess pain severity in unconstrained hospital environments using a decision-level fusion of 3 distinct types of features: data-driven RGB, entropy-based texture, and complementary features. They used 3 CNNs (VGG-CNN with transfer learning, entropy texture network, dual stream CNN) and various data augmentation techniques to avoid overfitting. The system demonstrates a 94.0% *F*_1_-score on a self-generated dataset from an unconstrained hospital setting.

Li et al [53] introduced a video-based infant monitoring system to analyze infant pain using 3 databases: Train-Data, Data-Clinic, and Data-YouTube. Using Fast Region-Based Convolutional Neural Network with object tracking and a hidden Markov model, the system precisely detects infant expressions and states. With a significant dataset from varied sources, including >16,000 images and real-world clinical videos, the approach offers enhanced accuracy and reliability in infant pain detection. Zamzmi et al [96] introduced a neonatal CNN that uses a cascaded architecture with 3 convolutional branches. This design merges image-specific and general information for pain detection. The neonatal CNN demonstrated 91% accuracy and 0.93 AUC on the Neonatal Pain Assessment Dataset and 84.5% accuracy on the Infant Classification of Pain Expression dataset. Witherow et al [97] developed Facial Expressions Fusing Betamix Selected Landmark Features (FACE-BE-SELF), a novel deep adaptive method for adult-child facial expression classification. It fuses facial landmark data with deep feature representations, achieving domain-invariant classification. Using a unique mixture of beta distributions, facial features are selected based on expression, domain, and identity correlations. The FACE-BE-SELF method stands out by concurrently adapting adult-child domains, providing a unified expression representation for both groups. Compared to standard approaches, it surpasses in aligning latent representations of expressions across age groups.

#### Vision-Based Spatiotemporal Features

Bargshady et al [98] present an ensemble deep learning model that combines a 3-stream hybrid neural network with CNNs to extract facial features and classify pain levels. The VGG-Face, integrated with principal component analysis (PCA), is used for early feature extraction, while a 3-layer hybrid of CNN and bidirectional LSTM is developed for late fusion classification. This approach, tested on multiple pain databases, surpasses competing models with an accuracy of >89%. Sparse Autoencoders for Facial Expressions-Based Pain Assessment [57] reconstructs the upper part of the face from input images and then feeds both the original and reconstructed images into 2 concurrent and coupled InceptionV3 using Sparse Autoencoders. This dual-input approach emphasizes the upper facial features, essential for pain detection. By eliminating the need for conventional preprocessing steps such as face detection and adeptly handling varying head poses, Sparse Autoencoders for Facial Expressions-Based Pain Assessment offers enhanced performance and accuracy across multiple datasets, even in challenging profile views. Karamitsos et al [94] modified temporal convolutional network algorithm and processed facial features extracted from fine-tuned VGG-Face and PCA combined with hue, saturation, and value color spaces. The temporal convolutional network–based approach showcases faster performance and higher efficiency, achieving an accuracy of 92.44% and an AUC of 85%. Bargshady et al [99] propose an enhanced joint hybrid CNN-Bidirectional LSTM network model by leveraging a fine-tuned VGG-Face for feature extraction and apply PCA to focus on the most significant features, improving computational efficiency. These features are then classified by a CNN-Bidirectional LSTM network hybrid network into 4 levels of pain intensity.

The 3D CNNs have gained attention in several studies. Tavakolian and Hadid [100,101] created a 3D CNN that captures dynamic facial representations from videos and emphasizes the typical use of a fixed temporal kernel depth in research, which often misses capturing different time ranges. In the study by Huang et al [102], a hybrid network by combining 3D, 2D, and 1D CNNs has been introduced to extract spatiotemporal, spatial, and geometric features from image sequences. Wang et al [103] used the convolutional 3D network for pain expression recognition, which primarily uses a 3×3×3 convolutional layer. However, this method often fails to capture the full spectrum of facial expression variations. To address this, they combined 3 distinct features: 3D CNN, HOG, and geometric features using support vector regression for pain estimation. They integrated the convolutional 3D network for spatiotemporal facial feature extraction and used the HOG in 2D images for geometric information to discern pain levels in facial expressions. De et al [104] present a deep learning architecture, the Decomposed Multiscale Spatiotemporal Network (DMSN). It uses 3 innovative blocks, DMSN-A, DMSN-B, and DMSN-C, to efficiently capture varied facial dynamics across conditions such as depression and pain. DMSN-A block focuses on pain, which might vary rapidly. It uses a sequence of 3×1×1 temporal convolutions, capturing short to long temporal ranges. The studies by Granger and Cardinal [105] and Praveen et al [106] implemented weak-supervised domain adaptation, focusing on a shift from general affective expressions to specific pain expressions. Their framework used an inflated 3D CNN [107] with 3 convolutional layers and 3 inception modules, extracting both spatial and temporal data from videos.

### Physiological Signals

#### Overview

While facial expressions are commonly used to identify pain, physiological signals are also a valuable modality for automatic pain detection. As detailed in the Pain Mechanism section, pain triggers changes in physiological signals, such as increased heart rate and skin conductivity, due to the activation of the SNS and peripheral nervous system [108]. Conversely, changes in physiological signals can indicate the presence of pain. However, extracting discriminative information from physiological signals is challenging. By contrast, they are objective indicators of pain because they cannot be artificially controlled [109], while exterior signals, such as facial expressions and gestures, may be unreliable, as individuals can deliberately disguise their behaviors. It makes physiological signals more reliable than exterior signals. In addition, physiological signals can be measured during daily life, while video and hand gestures can only be measured in laboratory settings. Thus, researchers have invested significant effort in exploring the feasibility of using physiological signals for pain assessment. Recent advances in sensor technology, signal processing, feature extraction, and ML algorithms are essential to the success of physiological signal–based automatic pain assessment.

This section provides a comprehensive review of the latest developments in pain detection approaches based on physiological signals. Four key components are exploited: (1) the use of modalities, (2) measurement devices, (3) feature extraction methods, and (4) ML models. The use of modalities refers to the type of physiological signals used for pain detection, including electroencephalogram, fMRI, electrocardiogram, and EDA. Measurement devices include both wearable and nonwearable devices, encompassing cardiac monitors, skin conductivity sensors, temperature sensors, accelerometers, and more. Feature extraction methods are techniques used to extract informative features from physiological signals, such as time-domain features, frequency-domain features, and time-frequency features. Finally, ML models, such as SVM, artificial neural networks, and random forest (RF), are used to classify pain based on the extracted features.

#### Electroencephalogram as a Pain Indicator

Electroencephalography is a noninvasive technique widely used in the automatic detection of pain. The electrodes detect electrical activity and amplify it, producing a graphical representation of the brain activity over time. Electroencephalogram recordings typically show a series of waveforms or oscillations that are grouped into different frequency bands, such as delta, theta, alpha, beta, and gamma. These frequency bands have been associated with different mental states and cognitive functions. Various studies have shown the potential of electroencephalogram-based pain detection, and different approaches have been proposed to extract discriminative features from electroencephalogram signals for pain classification. For instance, Panavaranan et al [110] extracted the power spectral density of an electroencephalogram using fast Fourier transform and used SVM to classify thermal pain. Hadjileontiadis et al [54] proposed a novel approach that analyzes wavelet higher-order spectral features of an electroencephalogram to predict tonic cold pain. Vijayakumar et al [111] extracted time-frequency wavelet representations of independent components from electroencephalogram data and trained a RF model to classify pain levels, achieving an intrasubject accuracy of 93.26%.

The use of electroencephalogram techniques for pain detection has great potential to provide objective measures of pain, as these methods directly measure brain activity related to pain perception. However, these techniques also have limitations, including high cost, limited availability, and the need for specialized expertise for data analysis.

#### fMRI as a Pain Indicator

fMRI is a powerful neuroimaging tool that measures changes in blood flow within the brain as a proxy for neural activity. By measuring changes in the blood oxygen level–dependent signal, fMRI can indirectly map changes in neural activity in response to a specific stimulus, such as a painful stimulus.

The fMRI technique has been widely used in pain research, revealing a network of brain regions that are activated by painful stimuli. These regions include the primary and secondary somatosensory cortex, thalamus, insular cortex, and anterior cingulate cortex, among others. The activation of these regions is believed to be involved in the sensory and affective components of pain processing.

Activation of these regions is thought to be involved in the sensory discrimination aspects of pain processing. Thus, neuroimaging techniques allow us to visualize and quantify brain activities and then quantify pain. It is frequently used in the research of automatic pain assessment. Wager et al [112] used the least absolute shrinkage and selection operator ML regression algorithm to recognize induced heat pain by assessing the fMRI activity patterns. Shen et al [60] derived primary, dorsal, and ventral visual networks from blood oxygen level–dependent fMRI scans by using independent component analysis and used a ML algorithm SVM to distinguish between patients with chronic low back pain and healthy volunteers and achieved an accuracy of 79.3%. Tu et al [59] proposed a novel sliced inverse regression–based fMRI decoding method to reduce the fMRI data dimension and showed overperformance compared to traditional regularization-based decoding analyses (principal component analysis and discriminant analysis, partial least squares-discriminant analysis, and least absolute shrinkage and selection operator). Robinson et al [58] scanned fMRI and applied ML algorithms to classify patients with fibromyalgia and healthy volunteers.

#### Electrocardiogram as a Pain Indicator

An electrocardiogram is a widely used technique to measure the electrical activity of the heart and its changes during each cardiac cycle. The electrocardiogram waveform consists of several characteristic waves and intervals that correspond to the different phases of the cardiac cycle, including the P wave, QRS complex, and T-wave. By analyzing the size, shape, and timing of these waves and intervals, a wide range of cardiac conditions, such as arrhythmias, heart attacks, and heart failure, can be diagnosed. The use of electrocardiograms in pain detection assumes that pain can cause a physiological stress response, leading to cardiovascular changes that are related to the pain stimuli. The autonomic nervous system responds to pain by increasing sympathetic tension and decreasing parasympathetic tension, leading to an increase in heart rate and blood pressure. By analyzing the electrocardiogram signal, features that reflect the autonomic nervous system status, such as heart rate variability (HRV), can be extracted and used to detect pain.

Several studies have shown the potential of electrocardiograms for pain detection. Walter et al [34] collected electrocardiogram data from 90 subjects using heat as pain stimuli and created the BioVid dataset, which also included skin conductance level, sEMG, and video data. Adjei et al [56] performed spectral analysis on electrocardiogram data and extracted HRV features, such as the low-frequency (LF) component and high-frequency (HF) component, which were significantly correlated with pain level. Jiang et al [64] extracted time-domain and frequency-domain HRV features from electrocardiogram data to classify pain level and obtained an AUC of 0.82 in the receiver operating characteristic curve.

However, there are also studies that suggest a lack of correlation between HRV and pain level. Meeuse et al [113] found no significant correlation between HRV features and heat pain level in their study. It is important to note that an electrocardiogram alone may not be sufficient to accurately detect pain, and other physiological signals, such as skin conductance and electromyography, may need to be considered as well. Furthermore, individual differences in pain perception and the variability of pain stimuli may affect the reliability of pain detection using an electrocardiogram.

#### EDA as a Pain Indicator

EDA, also referred to as galvanic skin response, is a physiological gauge of the skin’s electrical conductance. This conductance changes according to the functioning of sweat glands within the skin [114]. The measurement of EDA is a noninvasive process involving the placement of 2 electrodes, often on the fingers or palms. Activation of the SNS, triggered by situations such as stress or pain, leads to increased sweat gland activity, causing a rise in the skin’s electrical conductance.

Within the context of automated pain recognition, EDA serves as a valuable indicator due to its reflection of SNS activity [115], which is closely linked to the body’s response to pain. Numerous research studies have highlighted EDA’s potential in pain detection. For instance, in the BioVid dataset developed by Walter et al [34], EDA was used as one of the methods, revealing a correlation between EDA features and the intensity of pain.

sEMG is another important tool for measuring EDA in automatic pain detection. sEMG can measure the electrical activity of muscles and has been used to measure facial expression [116] or muscle movement of specific body parts, such as the back muscles [117]. These measures can provide additional information about the pain experience and may be used in combination with other modalities for better pain detection accuracy [118].

#### Devices

Data collection is indeed crucial in research, especially in statistical and ML-based studies. It is essential to ensure that the data collected are accurate, informative, and clean. However, selecting the right measurement devices is crucial for obtaining high-quality data.

**Figure 4:**
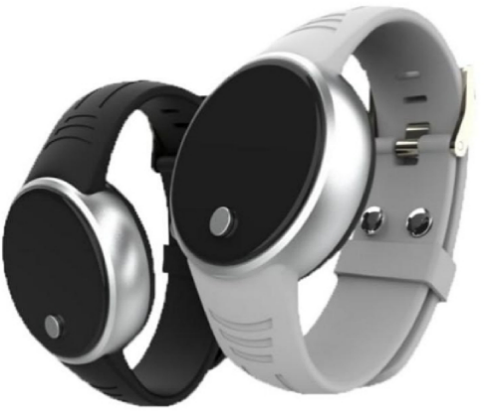
Devices used in physiological signal–based pain assessment: WeBe band.

Table 3 is a summary of previously used measurement devices in pain assessment studies. Figure 4 [115-117] presents 3 typical types of devices used in physiological signal–based pain assessment: wristband, headset, and chest band. The importance of wearable devices in this context cannot be overstated; they enable ubiquitous, real-time data collection [119,120], especially with the rise of body sensor networks. This technological advancement allows for extensive data gathering in wearable and remote settings, making continuous monitoring both feasible and affordable.

**Table 3:** Physiological signal measurement devices used in pain assessment studies.

Device	Physiological signals	Connectivity	Type	FDA^a^-cleared	Reference
Bioharness 3	Electrocardiogram	Bluetooth	Chest band	Yes	[64,69]
Affectiva Q sensor	EDA^b^	Bluetooth	Wristband	Yes	[68]
Procomp+	EDA and heart rate	Wired	Measurement hub	Yes	[121]
Emotive EPOC 14-channel electroencephalogram wireless recording headset	Electroencephalogram	Bluetooth	Headset	No	[54]
RespiBan	Respiration rate	Bluetooth	Chest band	No	[71]
Empatica E4	EDA, BVP^c^, and respiration rate	Wired	Wired sensor	Yes	[71]
Infiniti 3000A platform with Flex and Pro sensors	BVP, electrocardiogram, and EDA	Wired	Sensorhub	Yes	[65,67]
Polar RS800CX	HRV^d^	Wired	Watch	No	[122]

^a^FDA: Food and Drug Administration.

^b^EDA: electrodermal activity.

^c^BVP: blood volume pulse.

^d^HRV: heart rate variability.

There are several studies that have evaluated the usability and reliability of different measurement devices. Researchers can refer to these studies when choosing measurement devices for their own research. Ajayi et al [123] evaluated the Empatica E4 by comparing the results with nurse-recorded data and pooling questionnaires from participants. Nazari et al [124] tested the reliability of Bioharness and Fitbit measures of heart rate and activity at rest status. Rawstorn et al [125] evaluated the BioHarness by testing it on volunteers with both sinus rhythm and atrial fibrillation during simulated daily activities as well as low-, moderate-, and high-intensity exercises. Loberg et al [126] evaluated 4 different respiratory effort sensors and compared them with a respiratory sensor from NOX Medical as the golden reference device.

#### Feature Extraction

##### Overview

In the field of ML, pattern recognition, and image processing, feature extraction is a crucial step that involves transforming raw data into informative and nonredundant features to facilitate subsequent learning and generalization. Physiological signals typically carry implicit information that needs to be revealed through appropriate feature extraction techniques. While deep learning methods often generate features automatically, traditional ML methods require manual feature extraction.

For physiological signals, time window segmentation is commonly used to extract features. This involves segmenting the signals into chunks of equal time intervals and generating a row vector for each segment with 1 feature value for each feature, for example, the mean value of the segmentation. Physiological signal features can be classified into 4 categories: time-domain, frequency-domain, time-frequency-domain, and space-domain features.

Time-domain features describe the statistical and morphological properties of physiological signals, such as maximum value, SD, entropy, and mean R-R interval in electrocardiogram signals. Frequency-domain features characterize the spectral properties of signals, such as LF band power and low-high frequency ratio. Time-frequency-domain features consider both time-domain and frequency-domain properties simultaneously to account for the short duration and changing nature of physiological signals. Space-domain features, such as multispectral imaging and topography, are used to represent topographic characteristics of brain activity features, including electroencephalograms, fMRI, and fNIRS.

The complexity of physiological signals can guide feature selection. Signals with high stochastic stationarity and low signal-to-noise ratio, such as photoplethysmography and EDA, are considered low in complexity and can be represented by 1 or 2 feature domains. Signals with low stochastic stationarity and high signal-to-noise ratio, such as electrocardiogram, electroencephalogram, and fMRI, are high in complexity and require 3 to 4 feature domains to capture all relevant information. Nowadays, numerous Python libraries are available that facilitate the rapid extraction of features in physiological signals [127,128], electroencephalograms [129], video [130], and audio [131] domains. A summary of the commonly used features is presented in Table 4.

**Table 4:** Summary of the commonly used physiological signal features in pain assessment studies.

Category, feature, and description			Reference
	**HRV** ^a^ **time-domain measures**		[132]
		SD of NN^b^ intervals	
		SD of RR^c^ intervals	
		STD^d^ of the average NN intervals for each 5 min segment of a 24-hour HRV recording	
		Mean of the STD of all the NN intervals for 5-min segment of a 24-hour HRV recording	
		Percentage of successive RR intervals that differ by >50 ms	
		Average difference between the highest and lowest heart rates during each respiratory cycle	
		Root mean square of successive RR interval differences	
		Integral of the density of the RR interval histogram divided by its height	
		Baseline width of the RR interval histogram	
	**HRV frequency-domain measures**		[132]
		Absolute power of the ultra LF^e^ band (≤0.003 Hz)	
		Absolute power of the very-LF band (0.0033-0.04 Hz)	
		Peak frequency of the LF band (0.04-0.15 Hz)	
		Absolute power of the LF band (0.04-0.15 Hz)	
		Relative power of the LF band (0.04-0.15 Hz) in normal units	
		Relative power of the LF band (0.04-0.15 Hz)	
		Peak frequency of the HF^f^ band (0.15-0.4 Hz)	
		Absolute power of the HF band (0.15-0.4 Hz)	
		Relative power of the HF band (0.15-0.4 Hz) in normal units	
		Relative power of the HF band (0.15-0.4 Hz)	
		Ratio of LF to HF power	
	**HRV nonlinear measures**		[132]
		Area of the ellipse that represents the total HRV	
		Poincare plot SD perpendicular to the line of identity	
		Poincare plot SD along the line of identity	
		Ratio of SD1 to SD2	
		Detrended fluctuation analysis, which describes short-term fluctuations	
		Detrended fluctuation analysis, which describes long-term fluctuations	
		Correlation dimension, which estimates the minimum number of variables required to construct a model of system dynamics	
	**Amplitude**		
		Peak amplitude	[133]
		Peak to peak amplitude	[133]
		Root mean square	[134]
		Mean absolute value	[134]
		Mean relative time of the peaks	[135]
		Mean relative time of the valleys	[135]
	**Variability**		
		IQR	[135]
		Range	[133]
		SD	[133]
		Variance	[134]
		Mean resting rate	[132]
		Slope resting rate	[132]
	**Stationarity**		
		Integral degree of stationarity	[136]
		Modified integral degree of stationarity	[136]
		Modified mean degree of stationarity	[136]
		Median	[133]
		SD of SD vector	[133]
	**Entropy**		
		Approximate entropy	[137]
		Fuzzy entropy	[138]
		Sample entropy	[139]
		Shannon entropy	[140]
		Spectral entropy	[141]
	**Linearity**		[133]
		Lag dependence function	[136]
		Population lag dependence function	[136]
	**Similarity**		
		Correlation coefficient	[142]
		Median coherence	[143]
		Mean coherence	[143]
		Modified mean coherence	[143]
		Modified integral of coherence	[143]
		Mutual information	[144]
	**Frequency**		
		Bandwidth	[133]
		Center frequency	[133]
		Median frequency	[134]
		Mean frequency	[134]
		Mode frequency	[133]
		Zero crossings	[134]

^a^HRV: heart rate variability.

^b^NN: neural network.

^c^RR: 2 consecutive R waves.

^d^STD: SD.

^e^LF: low-frequency.

^f^HF: high-frequency.

##### Brain Activity Features

Physiological signals, including electroencephalograms, fMRI, and fNIRS, have unique characteristics that require specific feature extraction techniques. Electroencephalogram signals, for example, have high topological complexity as multiple channels are measuring simultaneously. They can be divided into different frequency bands, such as delta, theta, alpha 1, alpha 2, beta 1, beta 2, gamma 1, and gamma 2. To assess pain, Panavaranan et al [110] used power spectral density features calculated using fast Fourier transform. Hadjileontiadis et al [54] combined continuous wavelet transform with higher-order statistics and spectra to create a new feature space for electroencephalograms. Rissacher et al [55] found temporal parietal alpha of electroencephalograms to be a useful feature for pain assessment.

In fMRI, Tu et al [59] proposed a novel dimension reduction method by incorporating singular value decomposition into sliced inverse regression to overcome the limitations of sliced inverse regression when dealing with high-dimensional data. This method was used to assess pain, achieving 77.61% binary classification accuracy.

There are various feature extraction approaches for electroencephalogram signals, as summarized by Behzadfar et al [145]. For brain activity signals in general, van der Miesen et al [146] outlined the state and progress in pain detection using these signals.

##### Electrocardiogram Features

Unlike general statistical feature extraction methods, electrocardiogram feature extraction involves more human experience on electrocardiograms and is more interpretable. Shaffer et al [132] provided an overview of HRV features, covering time-domain, frequency-domain, and non-linear measures. Time-domain and frequency-domain features are widely used in pain assessment studies. On the BioVid dataset, Werner et al [147] derived mean resting rate, root mean square of successive differences, and slope resting rate from the electrocardiogram signal. Gruss et al [148], Campbell et al [149], and Kachele et al [150] used the same 3 features in their studies. Kachele et al [150] also applied 4-level wavelet decomposition on detected R peaks to extract the mean alpha 1 coefficients. Jiang et al [64] extracted time-domain features, such as average interval between normal heart beats, SD of normal heart beat intervals, root mean square of successive differences, and percentage of successive RR intervals that differ by more than 20 ms, and frequency-domain features, such as LF, HF, and LF or HF, from an electrocardiogram and attained an AUC of 0.82 for induced electrical pain and an AUC of 0.75 for induced thermal pain.

Apart from HRV, other features have been used for various purposes. For instance, some studies have used morphological features, such as QRS complex duration and amplitude, T-wave amplitude, and ST-segment changes, for diagnosing cardiac abnormalities [150].

##### EDA and Electromyography Features

EDA and electromyography are critical tools in pain detection because they measure physiological responses that are directly linked to the autonomic nervous system’s reactions, which vary significantly with pain perception [114,151]. Walter et al [133] systematically gathered and summarized feature extraction methods for EDA or electromyography signals from previous research and categorized them into mathematical groups of (1) amplitude, (2) frequency [152], (3) stationarity [136], (4) entropy [153], (5) linearity [144], and (6) variability. In total, 33 different features were listed, and their efficiency in pain assessment on the BioVid dataset was proved. Then, Gruss et al [148] deployed the feature table and derived it to 39 features. Campbell et al [149] also developed a feature list based on the study by Walter et al [133]. They also proposed a ML-based feature selection approach that deploys univariate feature selection and sequential forward selection for 100 epochs, with cross-validation as the metric to explore the optimal feature set. From their results, a relationship table between features and pain was displayed, illustrating the discriminative strength of features. In addition, amplitude, power, and unique functional features of electromyography signals are noted as useful in all different feature sets. Table 4 summarized the features used in previous studies.

#### Models

##### Overview

In the field of ML, the “no free lunch” theorem has been referred to often when talking about model selection [154]. This theorem illustrates that “any two optimization algorithms are equivalent when their performance is averaged across all possible problems,” which implies that no single algorithm always has the best performance for all ML tasks. Thus, appropriate model selection is necessary for the success of ML-based pain assessment. In this section, we compare different ML algorithms by illustrating their advantages and disadvantages and their applicable scenarios. Table 5 provides a summary of the prevalent ML algorithms used in pain assessment.

**Table 5:** Summary of the prevalent machine learning algorithms used in pain assessment studies.

Model	Advantages	Disadvantages	Reference
Support vector machine	Suitable for small datasets Takes advantage of kernel functions	Low performance in multiclass tasks	[64,71]
Decision tree	Easily interpretable Computation friendly	High risk of overfitting Discards correlations between features	[155]
Random forest	Applicable on large datasets Fixes the overfitting problem of decision tree Easy to parallelize	Low performance on low-dimensional datasets Time consuming	[156,157]
Neural networks	High performance with large amounts of data Flexible with layer configurations	Uninterpretable Computation consuming	[158,159]

##### SVM for Pain Classification

The first commonly used ML model in physiological signal–based automatic pain detection is SVM [64,71]. SVM is a type of generalized linear classifier that classifies data in a supervised learning way [160]. Its decision boundary is the maximum margin hyperplane for learning samples. SVM also includes kernel tricks, which makes it a substantially nonlinear classifier. The final decision of SVM only depends on the support vectors, which makes it suitable for small sample learning. On the contrary, SVM lacks the ability to provide restoration of variables to the formation of derived predictors [161], which is important in some areas such as financial prediction and health applications. In addition, SVM requires delicate preprocessing and tuning to acquire the best performance. Panavaranan et al [110] applied polynomial kernel SVM on electroencephalogram data and obtained an accuracy of 96.97%. Gruss et al [148] used SVM on the BioVid dataset and gained 90.94% accuracy on pain tolerance classification. In addition, Jiang et al [64] obtained an AUC of 0.82 with the use of SVM. More recently, Badura et al [71] achieved 94% accuracy using Gaussian kernel SVM.

##### Decision Tree for Pain Classification

Unlike SVM, decision tree is known for its interpretable characteristic. The decision tree algorithm is a method of approximating the value of a discrete function [162,163]. It is a typical classification method that uses an induction algorithm to generate readable rules and decision trees and then uses decision-making to analyze the new data. Essentially, a decision tree is a process of classifying data through a series of rules. Because of their inherent interpretability, tree-based algorithms help ML processes move beyond the “black box” model [164]. By contrast, due to the simple structure of tree-based models, overfitting easily happened on tree-based models [165]. Besides, they lack the ability to deal with missing data due to the continuity of tree structure.

##### RF for Pain Classification

RF is an algorithm that integrates multiple trees through the idea of ensemble learning. Its basic unit is a decision tree, and essentially, it belongs to a large branch of the ML “ensemble learning” method. Intuitively, each decision tree acts as a classifier, so for a given input sample, N decision trees will produce N classification results. RF integrates all classification voting results and designates the category with the most votes as the final output, which is a “bagging” idea. With the tree base and bagging theory RF holds, it has advantages such as preventing overfitting, easy to parallelize, and friendly with high-dimensional data [166]. In contrast, RFs require more time for training and prediction compared to decision trees. Vijayakumar et al [111] applied RF on 25 subjects’ electroencephalogram data and obtained 89.45% accuracy. Naeini et al [167] used RF on the BioVid dataset and achieved an accuracy of 79%. Werner et al [168] used RF on their new “X- ITE” dataset and achieved 94.3% accuracy for phasic electrical pain classification.

##### Neural Networks for Pain Classification

NN have also been used by scholars for automatic pain detection [158,159]. NN abstracts the human brain neuron network from the perspective of information processing, establishes a certain simple model, and composes different networks according to different connection structures. Thanks to the development of the digital society, the amount of data available for ML has grown substantially. NN, which can go deep in its layer structure, can reveal implicit information from data. Therefore, as the amount of data grows, the performance of NN keeps increasing, while traditional algorithms, such as SVM and RF, are limited. Nevertheless, NN has the defect of “black box” characteristic. Such uninterpretability keeps NN from blooming in certain fields, such as text and code analysis [169], judicial decision, and artificial intelligence medicine, because such fields require a clear, understandable, and interpretable decision-making process. Martinez et al [170] used NN on the BioVid dataset and obtained 82.75% accuracy on multitask classification. Jiang et al [69] applied an artificial neural network on 30 subjects and gained an average accuracy of 83.3%. The deviation of neural networks is widely used in automated pain assessment, such as CNN [156], RNN [171], and LSTM neural network [172].

### Audio Analysis

Infant crying is a common sign of discomfort, hunger, or pain. It conveys information that helps caregivers assess the infant’s emotional state and react appropriately. Crying analysis can be divided into two main stages: (1) the signal processing stage, which includes preprocessing the signal and extracting representative features; and (2) the classification stage. We classified the existing methods of signal processing stage into (1) time-domain methods; (2) frequency-domain methods; and (3) cepstral-domain methods.

Time-domain analysis is the analysis of a signal with respect to time (ie, the variation of a signal’s amplitude over time). Linear prediction coding is one of the most common time-domain methods for analyzing sounds. The main concept behind linear prediction coding is the use of a linear combination of the past time-domain samples to predict the current time-domain sample. Other time-domain features that are commonly used for infants’ sound analysis are energy, amplitude, and pause duration. Vempada et al [49] presented a time-domain method to detect discomfort-relevant cries. The proposed method was evaluated on a dataset consisting of 120 cry corpuses collected during pain (30 corpuses), hunger (60 corpuses), and wet diaper (30 corpuses). We want to note that the paper does not provide information about the stimulus that triggered the pain state or the data collection procedure. The infants’ age ranges from 12 to 40 weeks. All corpses were recorded using a Sony digital recorder with a sampling rate of 44.1 kHz. In the feature extraction stage, two features were calculated: (1) short-time energy, which is the average of the square of the sample values in a suitable window; and (2) pause duration within the crying segment. Part of these features were used to build SVM, and the remaining features were used to evaluate its performance. The recognition performance of pain cry, hunger cry, and wet diaper cry were 83.33%, 27.78%, and 61.11%, respectively. The average recognition rate was 57.41%.

### Pupil Size

The measurement of changes in pupil size has been shown to be a promising physiological indicator of pain intensity. Pupil size can be used to monitor the effects of painful stimuli in the brain. The pupil dilates in response to pain due to the activation of the sympathetic branch, which releases norepinephrine, and the inhibition of the parasympathetic branch, which is responsible for constriction of the pupil. This section discusses the mechanism of using pupil dilation as a pain indicator and literature reviews of using pupil dilation for automated pain assessment.

The pupil dilation is a complex physiological response regulated automatically by 2 muscles in the eye, the sphincter pupillae and the dilator pupillae. The sphincter pupillae is controlled by the parasympathetic system to contract the pupil, while the dilator pupillae is dominated by the sympathetic system to dilate the pupil [50].

Höfle et al [51] investigated the influence of different luminance conditions on pupillometry for pain detection and found that the baseline pupil size values significantly differed under different luminance conditions, while the peak dilation remained the same. Bertrand et al [173] explored the influence of gender and anxiety on pupil dilation for pain detection and concluded that pupil dilation changes similarly in both men and women and are exacerbated in the presence of anxiety. Connelly et al [52] conducted an experiment on 30 children undergoing elective surgical correction of pectus excavatum and found that maximum pupil size, percent change in pupil size, and maximum constriction velocity were the most related features to pain intensity. Chapman et al [174] reported a delay of 1.25 seconds in 20 adult volunteers under noxious stimulation, while Eisenacha et al [175] reported a peak in pupil size with a lag of 4.25 seconds after the onset of heat pain on 28 adult volunteers. Wang et al [176] found that the pupillary response together with ML algorithms could be a promising method of objective pain level assessment by measuring pupillary response during induced cold pain on 32 subjects.

### Multimodal Pain Detection

**Figure 5:**
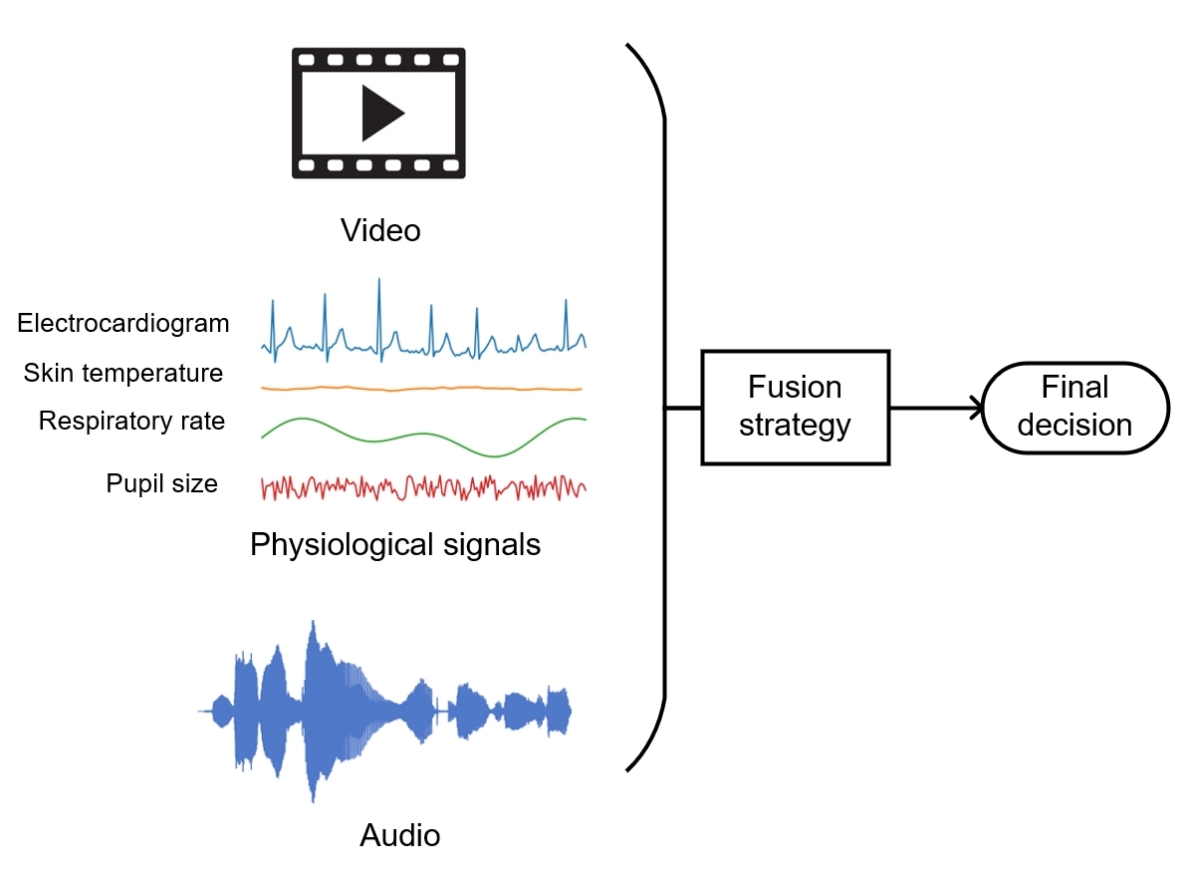
Multimodal pain assessment.

Including more modalities can possibly increase information density, which leads to increased accuracy. Thus, researchers have been increasingly turning to multimodal approaches to enhance the accuracy and reliability of automated pain assessment systems. These approaches combine information from multiple modalities, such as biomedical signals and facial expressions, to provide a more comprehensive understanding of the patient’s pain experience. Furthermore, a multimodal approach can capture a more nuanced and diverse range of pain responses, which is particularly important given the wide variation in pain perception among individuals with different characteristics and cultural backgrounds. Figure 5 presents a typical flow of multimodal pain assessment.

**Figure 6:**
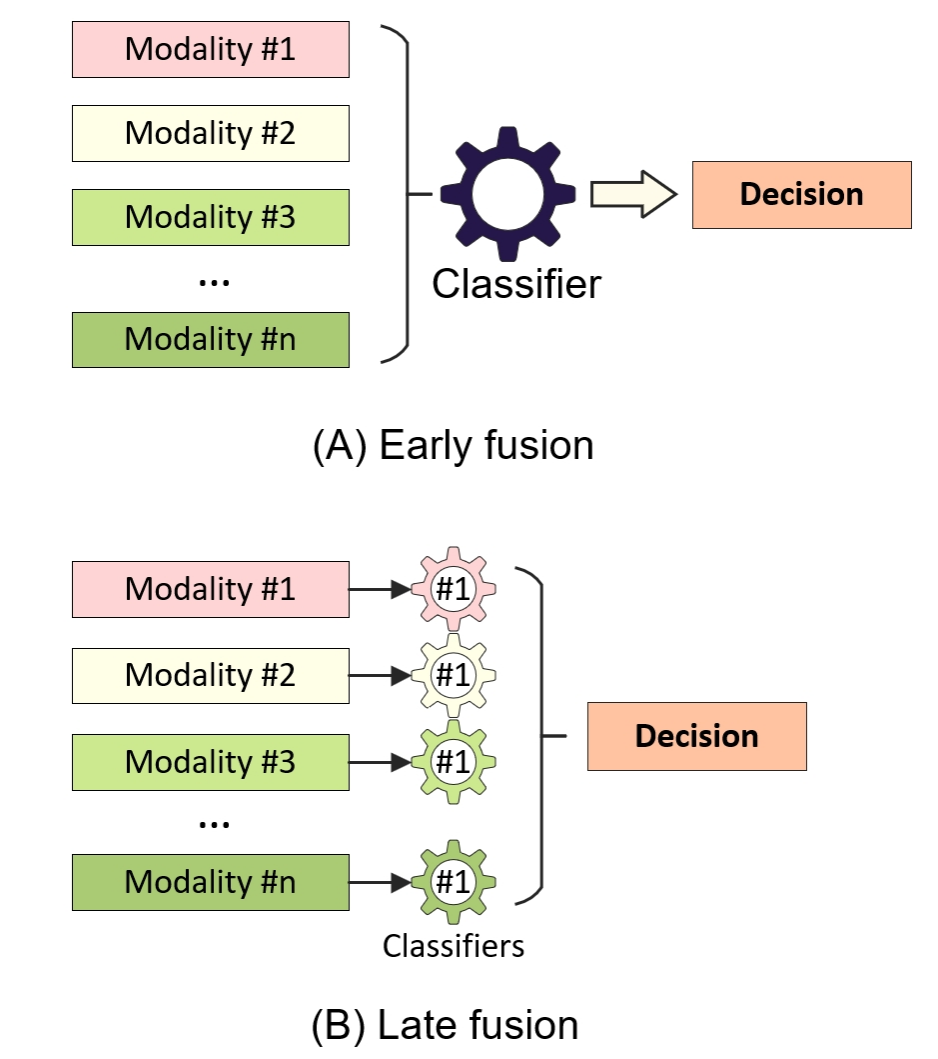
Fusion strategies.

Fusion strategies commonly used in multimodal pain assessment can be categorized into early fusion and late fusion. Early fusion involves the combination of features from different modalities before the training of a classifier, while late or decision fusion combines the predictions of individual classifiers after training. Common methods of combining predictions include fixed methods such as taking the mean or product and trainable methods such as using a pseudoinverse. Figure 6 illustrates the early and late fusion strategies. Some research has explored combining early and decision fusion by merging specific features at the feature level and then fusing those with other features at the decision level [46].

The first study to combine video and physiological signals for automated pain detection was conducted by Werner et al [147], who used an early fusion strategy to concatenate features from both modalities. The optimal fusion set is found to be the combination of all video and physiological signals, achieving accuracies of 80.6% and 77.8% for person-specific and generic classifiers, respectively, in detecting baseline and highest tolerable pain using a RF ensemble–based classifier. Kachele et al [177] applied both early and late fusion strategies using SVM with linear kernel and RF for recognizing baseline and highest tolerable pain, achieving accuracies of 68.2% and 76.6% for early and late fusion, respectively.

Continuing the BioVid dataset, Kachele et al [178] applies early and late fusion techniques with new features included, achieving slightly better results with late fusion (83.1%) than early fusion (82.7%). Thiam et al [179] proposed a hierarchical fusion architecture that divides multimodal data into 3 subsets. These subsets are used for the first layer of RF training, followed by pseudo-inverse mapping, multilayer perceptron mapping, and a final layer that combines both pseudo-inverse and multilayer perceptron fusion mapping. Kessler et al [180] took advantage of the fusion strategy proposed by Thiam et al [179] and applied it to remote photoplethysmography.

Other studies focus on incorporating additional modalities, such as audio. Velana et al [37] published the SenseEmotion database, which captures video, physiological signals, and audio for the first time. Thiam et al [181] merged features from video, physiological signal, and audio data on the SenseEmotion dataset, exploring different data fusion strategies, including early fusion, group late fusion, and individual late fusion. Results show that individual late fusion outperforms other strategies slightly on leave-subject-out experiment, while group late fusion slightly outperforms on user-specific task. There is also a dataset for neonatal pain assessment that includes video, audio, and physiological signals [46,171].

Recent studies have explored new fusion approaches. Bellmann et al [182] proposed a dominant channel fusion approach that identifies the most relevant input channel and combines it with the remaining channels to create an ensemble of classifiers. Bellman et al [183] proposed a novel late fusion approach that combines a mixture of experts and stacked generalization approaches and is assessed on different datasets involving the biophysiological modalities electromyography, electrocardiogram, and EDA. Thiam et al [159] proposed an information theoretic approach that uses a deep denoising convolutional autoencoder to learn and aggregate latent representations based on each input channel.

However, it is evident that late fusion, using multiple models as part of an ensemble learning approach, requires significantly more computational power and storage space compared to early fusion methods. As pain assessment is an emerging field, the current focus is predominantly on enhancing predictive accuracy rather than on resource use, and discussions on model complexity are relatively scarce. However, with the advent of Tiny ML and the rise of edge computing [184], running large models on microprocessors becomes challenging. Consequently, early fusion might gain popularity on edge devices, where the ability to run simpler, more compact models efficiently is crucial. This shift could make early and lightweight fusion approaches more viable and preferred in scenarios where computational resources are limited. In addition, with the increasing inclusion of multimodal data, we can envisage future fusion methods potentially incorporating recently developed self-attention algorithms [185].

## Discussion

The pain assessment field is faced with several challenges and opportunities for future development. This section will focus on 3 areas of concern—data, ML techniques, and ethical considerations—and then propose future research directions.

### Data

Automatic pain assessment is challenged by the limited availability of clinical pain data, as most studies have focused on experimental or induced pain. Widely used datasets such as BioVid, BP4D+, and X-ITE are collected from healthy volunteers and use external thermal or electrical pain. These studies are conducted under consistent experimental conditions that differ from real-world scenarios. Furthermore, induced pain has different mechanisms than disease pain, which encompasses different types of pain, such as nociceptive and central pain. Therefore, it is important to test models trained on experimental data using clinical pain data. In addition, more clinical pain data should be collected to facilitate the development of automatic pain assessment models and enable their use in clinical trials.

Pupil dilation has been identified as a promising indicator of brain activity and pain levels. However, in previous studies, pain was often used as the stimuli for measuring brain activity, rather than the focus of the study. Consequently, only a few studies have directly correlated pupil dilation with pain levels. A potential research direction is to include pupil dilation in the automatic pain assessment modality family. Pupil dilation has been shown to be effective in affective computing, with datasets such as the MAHNOB-HCI and SEED containing eye-tracking data that demonstrate the contribution of pupil data to arousal detection. As pain can also be regarded as physiological arousal, transferring pupil dilation to automatic pain assessment studies is a worthwhile area of research.

### Personalization of Pain Responses

In the following subsection, we explore personalized pain detection, focusing on the considerable differences in pain experiences among individuals. Pain perception varies widely due to a mix of biological factors and social-psychological influences. These differences are shaped by demographics such as gender, age, and ethnicity, which are linked to varying rates of chronic pain. In addition, factors such as genetic predispositions and psychological processes also significantly impact pain responses, whether in clinical settings or experimental scenarios. Importantly, these elements interact in complex ways, crafting the unique pain experiences of everyone. Research has highlighted that genetic markers associated with pain can differ across genders and ethnicities and interact with psychological aspects such as stress, affecting pain perception. These myriads of interacting factors culminates in a distinctive set of influences for each person’s experience of pain [186].

Jiang et al [187] introduced a method that enhances pain assessment by incorporating personalized features. They used ML to analyze individual pain data, enabling the model to tailor its predictions to each patient’s unique physiological and psychological characteristics. This approach improves the accuracy of pain management by adapting to personal pain profiles. Casti et al [188] developed a platform to improve pain diagnosis by leveraging personalized data. Using a combination of visual, speech, and physiological indicators, they used ML techniques to tailor assessments to individual patient profiles, enhancing the precision and effectiveness of pain management strategies. Martinez et al [189] proposed a method to refine pain estimation by integrating personalized features. They used ML to analyze individual facial expressions, allowing the model to adjust its predictions based on each person’s unique facial expressiveness score. This approach enhances the accuracy of Visual Analog Scale estimations by adapting to individual pain profiles [189].

Most papers on personalized pain assessment claim personalization at the model level, focusing on enhancing ML models to suit individualized approaches or using ML techniques to delve deeper into databases for extracting personalized information to improve predictions. The predominant reliance on public databases for research is evident, as most researchers use these readily available datasets. This reliance restricts personalization efforts to the data provided by these databases, making highly tailored training challenging. In addition, most pain-related datasets globally are derived from experiments involving artificially induced pain, which must pass rigorous ethical or clinical trial reviews, further limiting the quantity of available data. Looking to the future, personalization will undoubtedly be a crucial focus. It is foreseeable that researchers will collect more personalized data during experiments, including variables such as personality traits and ethnicity. This will likely lead to the generation of more nuanced datasets that include varied physiological responses to different pain stimuli, enhancing the granularity and effectiveness of personalized pain management solutions.

### Real-Time Pain Detection

Building on our earlier discussion about the personalization of pain responses, it is essential to delve into another critically relevant clinical application: real-time monitoring [190]. The goal of such monitoring is not just to detect pain but to enable timely and effective interventions that can significantly enhance patient outcomes. Real-time monitoring of pain becomes particularly crucial in postoperative care, where accurately gauging a patient’s pain levels is vital for adjusting analgesic dosages. This not only helps in managing the pain effectively but also minimizes the risk of both undermedication and overmedication, which can lead to complications such as opioid dependency or inadequate pain relief. In ICUs, the stakes are even higher. Many patients in ICUs are unable to communicate due to their conditions or sedation, making verbal reports of pain unreliable. Here, real-time monitoring systems can play a transformative role by continuously tracking pain indicators through physiological signals such as heart rate, blood pressure, and facial expressions. These data can then be analyzed to provide a dynamic, real-time assessment of pain, informing caregivers when an intervention is necessary. Moreover, real-time monitoring integrates seamlessly with the concept of personalized pain management. By continuously collecting and analyzing data specific to each patient, health care providers can tailor their interventions more precisely to the individual’s pain profile and response to treatment. This approach not only improves the quality of care but also enhances patient comfort and satisfaction. As technology advances, the potential for real-time pain monitoring grows. Innovations in wearable technology, ML algorithms, and data integration are paving the way for even more accurate and responsive pain management systems. These systems promise to transform how pain is managed in health care settings, making care more proactive, patient centered, and effective.

In the academic sphere, the development of real-time pain monitoring is primarily concentrated on 2 aspects: improving model efficiency to enable fast judgments suitable for real-time applications and developing practical tools such as wearable devices and mobile apps to facilitate widespread implementation. Enhancing the processing speed of models involves not only maintaining accuracy but also integrating advanced ML technologies, such as deep learning. Meanwhile, the development of tools such as wearables and mobile apps allows for the noninvasive collection of physiological data and real-time analysis, helping patients and health care providers to promptly assess pain levels and treatment effectiveness. This combination of improved models and practical tools is driving pain management toward more precise, personalized, and proactive solutions. Kong et al [191] introduced a smartphone app that enhances real-time pain detection using EDA signals collected from a wrist-worn device. They tested the app with thermal grill and electrical pulse data, demonstrating high accuracy in pain detection with a RF model. This approach offers a practical solution for objective, near–real-time pain assessment in everyday settings. Dai et al [93] address automatic pain detection using a mix of pain and emotion datasets to enhance model robustness, achieving 88.4% accuracy. They criticize CNNs for overfitting on biased data and validate their method through experiments on a humanoid robot in physiotherapy, emphasizing the importance of real-time, real-world testing and assessing the system’s practical utility and accuracy.

In summary, the advancement of real-time pain monitoring represents a significant enhancement in health care, enabling precise and timely interventions that are tailored to the unique needs of each patient. This technology not only improves the accuracy of pain assessments but also enriches the quality of care by integrating cutting-edge ML models and wearable technologies. As this field continues to evolve, it holds the promise of transforming pain management into a more responsive, personalized, and patient-centered practice.

### ML Techniques

Although deep learning has revolutionized computer vision and physiological signal analysis, traditional ML algorithms still dominate the field of physiological signal–based automatic pain assessment. One possible reason for this is that deep learning requires extensive data, which is time consuming and resource intensive to collect. Therefore, studies often include only a small number of participants, typically in the tens, making it difficult to gather comprehensive datasets.

In this context, transfer learning, a prominent topic in artificial intelligence, offers a promising alternative solution. Transfer learning involves applying knowledge gained from a source domain to a new target domain, which can be particularly useful in scenarios where data collection is challenging. Differing data distributions between the source and target domains can lead to performance degradation if models are applied directly. Transfer learning helps bridge this gap, ensuring better model performance across different settings [192].

Kächele et al [193] proposed an adaptive confidence learning method for personalizing pain intensity estimation systems, demonstrating the efficacy of transfer learning in this field. Feature extraction involved specific preprocessing steps for each signal type, such as bandpass filtering and artifact correction for electromyography. A multistage ensemble classifier was applied to learn the confidence of a regression system. This method involved selecting confident samples from unlabeled data of the test participants to iteratively adapt the model. Their experiments showed that the adaptive learning approach significantly improved the performance of pain intensity estimation.

Chen et al [194] implemented “TrAdaboost,” a transfer learning algorithm, to improve facial expression recognition, including pain expressions. They used the PAINFUL database, which contains video sequences of 25 patients with shoulder injuries, encompassing 48,398 frames of spontaneous pain expressions. The primary challenge addressed was the variability in pain expressions across different individuals. They proposed an inductive transfer learning algorithm to develop person-specific models. This algorithm first trains a set of weak classifiers on source data from multiple subjects and then selects the most relevant classifiers for the target subject. Experimental results showed that inductive transfer learning significantly improved pain detection accuracy. For example, the AUC for pain detection increased from 0.769 to 0.782 with just 10 target samples and reached 0.891 with 100 samples. Furthermore, this approach drastically reduced training time compared to traditional methods, making it feasible for rapid retraining in clinical settings.

While traditional ML remains prevalent in automatic pain assessment due to data constraints, transfer learning presents a viable alternative. It addresses the challenges associated with varying data distributions and limited dataset sizes, enhancing model robustness and performance. Future research should explore the potential of transfer learning algorithms further, integrating them into clinical practice to improve pain management outcomes.

### Ethical Considerations

Automatic pain assessment raises several ethical concerns that need to be addressed. One primary concern is the privacy and security of patients’ health data. The use of physiological signals, such as facial expressions, speech patterns, and pupil dilation, to assess pain levels can lead to the collection of sensitive health data. Therefore, it is essential to ensure that the data collected are secure and protected from unauthorized access.

Another ethical consideration is the potential for bias in automatic pain assessment models. ML models are only as good as the data they are trained on, and if the training data are biased, the model will be biased too. Bias can result in inaccurate pain assessment, leading to inadequate pain management and, in some cases, even harm to patients. Therefore, it is crucial to ensure that the data used to train the models are representative and unbiased.

### Future Directions

Automated pain assessment has made significant strides in recent years, leveraging technological advancements and data-driven approaches to enhance the accuracy and efficiency of pain detection. However, several promising directions for future research remain unexplored. Addressing these areas could lead to the development of more sophisticated and reliable automated pain assessment systems.

First, integrating data from various sources, such as pupil dilation, voice analysis, and body movement, could offer a more comprehensive understanding of pain. This requires a more comprehensive, clinical, and clean database to be released. Second, exploring novel deep learning architectures, including transformer-based models and generative adversarial networks, may yield improved performance in pain assessment tasks. These architectures could capture intricate patterns and dependencies within pain-related data, leading to enhanced predictive capabilities. Third, collaboration with health care professionals is crucial to validate the effectiveness and reliability of automated pain assessment systems in real-world clinical settings. Integrating these systems into clinical workflows could provide valuable insights and assist health care providers in making informed decisions. Finally, using transfer learning can provide new insights. In scenarios where large, annotated datasets are scarce, exploring transfer learning techniques and methods to adapt models to smaller datasets could prove beneficial. These approaches could enable the development of accurate pain assessment models even with limited training data.

### Conclusions

This survey reviewed the current advancements in automated pain assessment using ML techniques. Traditional pain assessment methods, reliant on self-reports and observational scales, face significant limitations, particularly for patients who are noncommunicative. We explored various modalities for automated pain detection, including facial expressions, physiological signals, audio, and pupil dilation. While each modality has its strengths, combining multiple modalities can enhance accuracy but also introduces challenges in data fusion and model complexity. Despite progress, challenges remain, such as the scarcity of diverse clinical pain datasets and ethical concerns regarding patient privacy. Personalized pain assessment models are also necessary due to variability in pain perception across populations. Future research should focus on developing more robust algorithms and leveraging deep learning and transfer learning. Collaborative efforts to create comprehensive pain datasets are crucial, as is integrating real-time pain monitoring into clinical practice. In summary, automated pain assessment has the potential to transform pain management. Continued interdisciplinary research and collaboration are key to overcoming current challenges and fully realizing these technologies’ benefits.

## Appendix

Multimedia Appendix 1 Summary of studies table.

## Abbreviations

AU: action unit
AUC: area under the curve
B-CNN: bilinear convolutional neural network
CNN: convolutional neural network
DMSN: Decomposed Multiscale Spatiotemporal Network
EDA: electrodermal activity
FACE-BE-SELF: Facial Expressions Fusing Betamix Selected Landmark Features
FACS: Facial Action Coding System
fMRI: functional magnetic resonance imaging
fNIRS: functional near-infrared spectroscopy
HF: high-frequency
HOG: histogram of oriented gradients
HRV: heart rate variability
ICU: intensive care unit
LBP: local binary pattern
LF: low-frequency
LSTM: long short-term memory
ML: machine learning
PCA: principal component analysis
RF: random forest
RGB: red, green, blue color model
RNN: recurrent neural network
RVR: relevance vector regression
sEMG: surface electromyogram
SNS: sympathetic nervous system
SVM: support vector machine
