# Supplementary material for: Survey on Pain Detection Using Machine Learning Models: Narrative Review
Source: JMIR AI. 2025 Feb 24;4:e53026. doi: 10.2196/53026 (PMC11894359; doi:10.2196/53026)
Supplement: Multimedia Appendix 1 [file ai_v4i1e53026_app1.pdf]

Title	Dataset	Stimulus	Modalities	Label	Features	Algorithms	Results
(2007) ICA-based ECG removal from Surface Electromyography and its effect on Low Back Pain Assessment	10 healthy subjects and 10 LBP[1] subjects	Back surgery	sEMG[2]	Binary[3]	Median frequency	Pearson correlation	ICA-denoised sEMG showed significant higher median frequency than raw sEMG
(2007) Identifying Frequency-Domain Features for an EEG-Based Pain Measurement System	15 healthy subjects	Cold	EEG[4]	N/A	FFT[5] components from Delta, Theta, Alpha1, Alpha2, Beta1, Beta2, Gamma1, Gamma2 bands	Pearson correlation	The temporal parietal alpha may be a useful feature for pain assessment.
(2008) Skin conductance compared to a combined behavioural and physiological pain measure in newborn infants	32 infants	Heel-lancing	Pulse rate SpO2[6], GSR[7]	Binary	Baseline level, wave change, amplitude change, PIPP score[8]	Pearson correlation and ROC[9]	0.815 AUC[10] of ROC
(2010) Skin Conductance Fluctuations Correlate Poorly with Postoperative Self-report Pain Measures in School-aged Children	90 school-aged[11] chilren	Heat	SCL[12]	Self-report pain level	Deviation between two sampling intervals	Spearman correlation and ROC	94.5% data showed an increase in SCL with pain induced. 0.691 of AUC of ROC
(2011) Autonomic responses to heat pain: Heart rate, skin conductance, and theirrelation to verbal ratings and stimulus intensity	39 healthy subjects	Heat	SCL, HR	N/A	SCL, HR[13]	N/A	r=0.46, p=0.01 at 46.5°C for HR
(2013) EEG-based Pain Estimation via Fuzzy logic and polynomial kernel Support Vector Machine	9 healthy subjects	Heat	EEG	Binary	Power spectral density	SVM[14]	96.97% accuracy
(2013) Heart Rate Variability Parameters Do Not Correlate with Pain Intensity in Healthy Volunteers	73 healthy subjects	Heat	ECG[15]	N/A	IBI, SDNN, LF, HF, LF/HF[16]	Pearson correlation	ECG features do not show significant correlation with pain
(2013) Towards Pain Monitoring: Facial Expression, Head Pose, a new Database, an Automatic System and Remaining Challenges	BioVid	Heat	Video	Cross test[18]	Frame level facial expression features	SVM	60%-80% accuracy depends on age
(2014) Automatic Pain Recognition from Video and Biomedical Signals	BioVid	Heat	Video, ECG EMG(*3), SCL (early fusion)	Cross test	GSR: amplitude and variability features, statistical features and stationary features. EMG: mode and mean frequency, width and central, median frequencies of the band and the count of zero-crossings. ECG: mRR, RMSSD[19], Slope of linear regression	RF	76% accuracy for pain tolerance
(2014) Physiological Signals Based Quantitative Evaluation Method for the Pain	6 healthy subjects	Electrical	BVP[20], ECG, SCL	Cross test with 6 pain levels	10 features from ECG (mean, std, etc) 12 features from SCL (diff-mean, etc) 18 features from BVP (diff-range, etc) with dimensionality reduction methods PCA-FP[21]	LDA[22]	98% accuracy for pain tolerance
(2014) Automatic pain quantification using autonomic parameters	BioVid	Heat	EMG, ECG, GSR	Cross test	A total of 135 features from biosignals and are selected by forward selection and backward selection	SVM with rbf kernel	59.44-81.75% accuracy for forward selection on individual test, 52.41-74.59% accuracy for general leave-subject test
(2015) Bio-Visual Fusion for Person-Independent Recognition of Pain Intensity	BioVid	Heat	Video, ECG EMG(*3), SCL (early and late fusion)	Cross test	131 features including: EMG, SCL: peak height, peak difference, etc ECG: RMSSD, RR difference, Slope of linear regression	SVM and RF[23]	77.2% accuracy for SVM 78.9% accuracy for RF
(2015) Multimodal Data Fusion for Person-Independent, Continuous Estimation of Pain Intensity	BioVid	Heat	Video, ECG EMG, SCL (early and late fusion)	Binary classification and regression	840 dimensional geometric-based features and appearance-based features	RF for classification and support vector regression	83.1% accuracy on pain tolerance using late fusion RMSE[24] of 0.98 on regression task
(2015) EEG-Based Tonic Cold Pain Characterization Using Wavelet Higher Order Spectral Features	17 healthy subjects	Cold	EEG	Binary	Wavelet higher order spectral features	QDA[25], mahalanobis (MAH), k-NN[26], SVM	90.25% accuracy
(2015) Pain Intensity Recognition Rates via Biopotential Feature Patterns with Support Vector Machines	BioVid	Heat	ECG,EMG(*3),SCL	Cross test	159 in total from amplitude, frequency, stationarity, entropy, linearity, variability and similarity aspects.	SVM	90.94% accuracy for pain tolerence and 79.29% accuracy for pain threshold.
(2016) Adaptive confidence learning for the personalization of pain intensity estimation systems	BioVid	Heat	Video, ECG EMG(*3), SCL (early and late fusion)	Cross test	ECG: amplitudes of P, Q, R, S and T points, the time delay between points and the angles of the Q and S valleys and the mean of approximation coefficients. EMG: 34 features including mean, std, etc. SCL: 18 features including skewness, kurtosis, etc	NN[27] ensembled with RF	83.1% accuracy for pain tolerence
(2016) Methods for Person-Centered Continuous Pain Intensity Assessment From Bio-Physiological Channels	BioVid	Heat	EMG, ECG, SCL	Cross test	EMG: 36 features from time and frequency domains ECG: 12 features including amplitudes of PQRST complexes, time delay, etc SCL: 18 features including skewness, kurtosis, etc	Personalized method with RF	40.1% accuracy for multiclass tasks.
(2016) Short-term assessment of autonomic nervous system as a potential tool to quantify pain experience	16 healthy subjects	Bending down	HRV[28]	Binary	HR, SD, RMSSD	Pearson correlation	p-value: 0.067 for HR 0.09 for SD 0.089 for RMSSD
(2017) Multi-task neural networks for personalized pain recognition from physiological signals	BioVid	Heat	ECG,SCL	Cross test	12 features from SCL including max, range, etc 5 features from ECG: RR interval, RMSSD, SDNN, LR Slope[29], SDNN/RMSSD	NN	82.75% accuracy for Multi-task NN

1. Low back pain, 2. surface electromyographic, 3. binary classification between pain and no pain, 4. electroencephalogram, 5. fast Fourier Transform, 6. percentage of oxygen in one's blood, 7. galvanic skin response, 8. premature infant pain profile, 9. receiver operating characteristic, 10. area under curve, 11. aged 7-17, 12. skin conductivity level, 13. heart rate, 14. support vector machine, 15. electrocardiogram, 16. interbeat interval, standard deviation of the IBI, low frequency components, high frequency components, LF/HF, 17. EMG at trapezius, corrugator and zygomaticus muscle, 18. cross test between baseline versus each pain level, in BioVid there are four pain levels, 19. mean RR interval, root meansquare of successive differences between normal heartbeats, 20. blood volumn pulse, 21. principal component analysis-fisher projection, 22. linear discriminant analysis, 23. random forest, 24. root mean squared error, 25. quadratic discriminant analysis, 26. k-nearest neighbors, 27.neural networks, 28. heart rate variability, 29. linear regression slope.

TABLE I: Summary of studies

Title	Dataset	Stimulus	Modalities	Label	Features	Algorithms	Results
(2017) Pain Prediction From ECG in Vascular Surgery	16 patients	Vascular surgery	ECG	Self-report pain level	LF, HF powers, LFPE[1], HFPE values.	linear regression	Kappa coefficient: 0.72 AUC of ROC: 0.97
(2017) Multi-modal Data Fusion For Pain Intensity Assessment and Classification	SenseEmotion	Heat	Audio, Video, EMG RSP[2], ECG, GSR (early and late fusion)	Cross test	Audio: 32 low level features and 14 high level features Video: Geometric descriptors, head pose descriptors and appearance-based descriptors Biosignals: time and frequency domain features	Both early fusion and late fusion are tested with random forest as base classifier	83% accuracy for user independent classification and 85% accuracy for user specific classification
(2017) Physiological signal-based method for measurement of pain intensity	6 healthy subjects	Electrical	BVP, ECG, SCL	Cross test	12 features (mean, min, max, range, std, etc) for each modality	SVM	96.47% accuracy
(2017) Quantifying and Characterizing Tonic Thermal Pain Across Subjects From EEG Data Using Random Forest Models	25 healthy subjects	Heat	EEG	Cross test with 4 pain levels	60 features from time-frequency representations	RF	89.45% accuracy
(2017) Ultra-Short-Term Analysis of Heart Rate Variability for Real-time Acute Pain Monitoring with Wearable Electronics	30 healthy subjects	Heat and electrical	ECG	Cross test with 3 pain levels	Time domain: AVNN, SDNN, RMSSD, pNNx[3] frequency domain: LF, HF, LF/HF	SVM	AUC of 0.82 time window of 40-50s is the best for electrical pain and AUC of 0.75 with 60s time window is the best for thermal pain
(2018) Automated Pain Assessment using Electrodermal Activity Data and Machine Learning	21 teenagers	Surgery	EDA[4]	Binary	TSD[5] features	PCA, linear SVM	77.66% accuracy
(2018) Continuous Pain Intensity Estimation from Autonomic Signals with Recurrent Neural Networks	BioVid	Heat	ECG, GSR	Cross test and regression	ECG: 7 features in time and frequency domain GSR: 6 features in time domain	RNN[6] regression and traditional ML	Classification: 74.21% accuracy on tolerance pain Regression: 1.05 MAE[7] using LSTM-NN[8]
(2018) Deep Multimodal Pain Recognition: A Database and Comparison of Spatio-Temporal Visual Modalities	MIntPAIN	Electrical	Video	5-class classification	Fc7[9] layer of the fine-tuned VGG-FACE[10] model	CNN[11]+LSTM	36.55% accuracy
(2019) Acute pain intensity monitoring with the classification of multiple physiological parameters	30 healthy subjects	Heat and electrical	HR, RSP, GSR, fEMG[12]	Cross test with 3 pain levels	Root mean squre and wavelength of fEMG, HR, RSP, GSR	NN	average accuracy of 83.3%
(2019) An Edge-Assisted and Smart System for Real-Time Pain Monitoring	BioVid	Heat	ECG,EMG(*3),SCL	Cross test	156 features from time, frequency domains	SVM and RF	no pain v.s. pain level one: 79% accuracy
(2019) Feature Extraction and Selection for Pain Recognition Using Peripheral Physiological Signals	BioVid	Heat	ECG,EMG(*3),SCL	Cross test	39*4 (for EMG and SCL) and 3*1 ECG features	Linear SVM	90% accuracy for pain tolerence
(2019) Twofold-Multimodal Pain Recognition with the X-ITE Pain Database	X-ITE	Thermal, electrical, phasic, tonic	Video, Audio, EMG, ECG, EDA (late fusion)	Cross test	1500+ dims feature space	RF	83.3% accuracy for phasic heat pain 94.3% accuracy for phasic electrical pain 86.6% accuracy for tonic heat pain 89.5% accuracy for tonic electrical pain
(2019) A Deep Neural Network-Based Pain Classifier Using a Photoplethysmography Signal	100 patients subjects	Post-opearation	PPG	Binary	PPG: 12 time domain features 6 frequency domain features	MLP[13], SVM and DBN[14]	86.79% accuracy using DBN
(2019) Exploring Deep Physiological Models for Nociceptive Pain Recognition	BioVid	Heat	EMG, ECG, GSR	Binary	N/A	CNN with raw data input	84.4% accuracy
(2020) Multimodal Deep denoising convolutional autoencoders for pain intensity classification based on physiological signals	BioVid	Heat	EMG, ECG, GSR	Binary classification and regression	N/A	CNN+LSTM	94.12% accuracy for tolerance pain
(2021) Multimodal Signal Analysis for Pain Recognition in Physiotherapy Using Wavelet Scattering Transform	34 subjects	Therapy	EDA, EMG, RSP, BVP, GRIP (early fusion)[15]	Cross test with 3 pain levels	20 wavelet scattering transform features and 13 statistical features	SVM, Adaboost	severe v.s. no-pain analysis reveals 94% and 82% accuracy for SVM and Adaboost, respectively
(2021) Multi-Modal Pain Intensity Assessment Based on Physiological Signals: A Deep Learning Perspective	BioVid & SenseEmotion	Heat	EMG, ECG, GSR	Cross test and multi-class task	Automatically generated features from deep neural networks	Deep denoising convolutional auto-encoder embedded with attention mechanism, trained with self-supervised learning	BioVid: 84.25% on BL v.s. PAt[16] 35.44% on 5 classes SenseEmotion: 81.05% on BL v.s. PAt 40.77% on 4 classess
(2021) Comparison of Feature Extraction Methods for Physiological Signals for Heat-Based Pain Recognition	BioVid & PainMonit	Heat	GSR	Cross test	Methods:HCF, dPhEDA, TVSymp, MTVSymp, MLP, CNN, LSTM and CAE[17]	RF, MLP	BioVid: 78.46-81.66% PainMonit: 79.44-84.71% accuracy for BL v.s. PL4

1. low frequency permutation entropy, 2. respiration rate, 3. average of NN intervals, standard deviation of NN intervals, root means square of successive differences between normal heartbeats, the fraction of consecutive NN intervals that differ by more than x ms, 4. electrodermal activity, 5. timescale decomposition, 6. recurrent neural networks, 7. mean absolute error, 8. long short-term memory networks, 9. fully connected layer, 10. a series of models developed for face recognition and demonstrated on benchmark computer vision datasets, 11. convolutional neural networks, 12. facial EMG, 13. multilayer perceptron, 14. deep belief network, 15. hand force, 16. pain tolerance.

TABLE II: Summary of studies (continued)

Title	Dataset	Stimulus	Modalities	Label	Features	Algorithms	Results
(2021) Multimodal spatio-temporal<br>deep learning approach for neonatal<br>postoperative pain assessment	USD-MNPAD-I	Procedural/postoperative	Audio, Video (early fusion)	Binary	Facial video: VGG-net<br>Audio: MFCC[1]<br>Movement: Motion image and VGG-16	KNN, RF<br>VGG-16 model + RNN	78.95% accuracy<br>0.8791 AUC of ROC
(2021) Multi-Modal Pain Intensity Recognition<br>Based on the SenseEmotion Database	SenseEmotion	Heat	EMG, RSP, ECG, GSR	Cross test	Physiological signals:<br>65 common feature for each modality	Early fusion, late fusion<br>with grouped modalities and<br>individual modalities embedded<br>with random forest classifier	84.72% accuracy on BL v.s. PA3<br>on user specific test and<br>83.95% accuracy on BL v.s. PA3<br>on user independent test
(2022) An Automatic System for<br>Continuous Pain Intensity Monitoring<br>Based on Analyzing Data from<br>Uni-, Bi-, and Multi-Modality	X-ITE	Heat and electric<br>phasic and tonic pain	Video, Audio, EMG<br>ECG, GSR<br>(early fusion)	Binary and multi-class	Video: 3 head poses, 17 AU intensity<br>features Audio: 4 energy features,<br>6 voicing features and<br>14 spectral features<br>ECG: RR intervals<br>EMG<br>GSR: filtered data	Random forest baseline<br>LSTM and LSTM with<br>sample weighting	Classification (binary):<br>Uni-modality: 79.8%<br>Bi-modality: 80.5%<br>Multi-modality: 79.3%<br>Regression:<br>Uni-modality: 0.07 MSE (GSR)<br>Bi-modality: 0.07 MSE (GSR+EMG)<br>Multi-modality: 0.05 MSE
(2022) Machine Learning-Based Pain Intensity<br>Estimation: Where Pattern Recognition<br>Meets Chaos Theory	BioVid & MFeat	Heat	EMG, ECG, GSR	Cross test	Fourier coefficients: 76 features<br>Profile correlations: 216 features<br>Karhunen-Loeve coefficients: 64 features<br>Pixel averages in 2*3<br>Zernike moments: 47 features<br>Morphological features: 6 features	Decision tree	$\Delta Nds : 0.19 and 0.25$<br>$\Delta Acc : 0.14 and 2.5$<br>$\Delta Out : 0.27 and 3.67$
(2022) Personalized Deep Bi-LSTM RNN Based Model<br>for Pain Intensity Classification<br>Using EDA Signal	Self-collected with<br>29 subjects	Cold	GSR	Cross test with<br>3 pain levels	Phasic and tonic<br>component features of GSR	Deep recurrent neural<br>networks based model<br>BiLSTM-XGB[2] model	BiLSTM RNN: 0.84 F-1 score<br>Multi-class: 0.81 F-1 score
(2022) Automatic detection of pain<br>using machine learning	41 healthy subjects	Cold	Respiration, HRV	binary	Respiratory features and HRV time,<br>frequency domain features	Logistic regression classifier	(1) laboratory/clinical use with<br>an F1 score of 81.9% and<br>(2) field/ambulatory use<br>with an F1 score of 79.4%
(2022) Automatic pain assessment on cancer patients<br>using physiological signals<br>recorded in real-world contexts	21 cancer patients	cancer pain	PPG, EDA, skin temperature<br>and accelerometer	Binary	12 HRV time and frequeancy<br>domain features, 5 PPG morphological<br>analysis features,<br>17 EDA features,<br>3 temperature features<br>and 2 ACC features	SVM, RF, MLP,<br>logistic regression<br>and Adaboost	72% accuracy
(2022) Experimental Exploration of Objective<br>human pain assessment<br>using multimodal sensing signals	28 healthy subjects	Heat	Video, EEG, eye movement,<br>EDA, BVP, EMG, Respiration,<br>skin temperature, BP<br>(early fusion)	Three-class<br>classification	Intuitive feature extraction<br>with ANOVA[3]	Cascade SVM based<br>classification tree	Results showed that the<br>use of all modalities yielded<br>highest results while<br>the use of only<br>EEG and video yield<br>second best result.
(2022) Accurate classification of pain experiences<br>using wearable electroencephalography in adolescents<br>with and without chronic musculoskeletal pain	39 healthy subjects and<br>121 mostly female pediatric<br>chronic pain patients	Cold	EEG	Binary	Seven classes of EEG features<br>were calculated for each<br>10-second time window:<br>spectral power, peak frequency,<br>permutation entropy, weighted<br>phase lag index,<br>directed phase lag index,<br>graph theory features in<br>four frequency bands.	SVM, logistic regression	75.2% accuracy in chronic pain<br>group and 74.8% accuracy<br>in control group.
(2023) Explainable Artificial Intelligence (XAI)<br>in Pain Research: Understanding the Role of<br>Electrodermal Activity for Automated Pain Recognition	BioVid, PainMonit	Heat	EDA	Binary	Hand-crafted EDA features	Random Forest, MLP,<br>CNN, CAE[4], Supervised CL,<br>Transformer, MDK-Resnet[5]	Results showed random forest<br>yielded best performance on<br>PainMonit dataset (91.70% accuracy)<br>while supervised CL yielded<br>best performance on<br>BioVid dataset (84.54% accuracy).
(2023) Design and Evaluation of Deep Learning<br>Models for Continuous Acute Pain Detection<br>based on Phasic Electrodermal Activity	BioVid, ChonLab thermal<br>grill pain database	Contrasting temperature	EDA	Binary	Phasic Eda, phasic<br>driver, TFS-phEDA[6]	LSTM and CNN<br>and hybrid architectures	The parallel TCN-SBU-LSTM[7] was<br>the best model when using<br>phasic driver and TFS-phEDA,<br>obtaining F1-score of 0.778.
(2023) PainVision-based evaluation of brain<br>potentials: a novel approach for<br>quantitative pain assessment	14 healthy subjects	Electrical	EEG	N/A	P2, N2 latency,<br>P2, N2, P2-N2 amplitude	N/A	Results showed P2 amplitude<br>can significantly discriminate<br>pain and no pain.
(2023) Personalized and adaptive neural networks<br>for pain detection from multi-modal<br>physiological features	BioVid & SpaExp	Heat, electrical	ECG, GSR	Binary and five-class<br>classification	15 features from EDA phasic driver,<br>tonic component, phasic component<br>and ECG time and frequency domain	Base neural networks with<br>dynamic feature attention<br>module and<br>personalization module	Results showed with pre-training<br>using SpaExp database<br>and training layers but<br>first two with BioVid yielded<br>promising results of 84.58% accuracy.

1. mel frequency cepstral coefficient, 2. bidirectional long short-term memory-Xgboost, 3. analysis of variance, 4. convolutional autoencoder, 5. multi-dilated kernel (MDK) residual network, 6. time-frequency spectrum of the phasic EDA, 7. temporal convolutional network-stacked bi-directional and unidirectional LSTM.

TABLE III: Summary of studies (continued)

References

[1] J. N. Mak, Y. Hu, and K. Luk, "Ica-based ecg removal from surface electromyography and its effect on low back pain assessment," in *2007 3rd International IEEE/EMBS Conference on Neural Engineering*. IEEE, 2007, pp. 646–649.

[2] D. Rissacher, R. Dowman, and S. Schuckers, "Identifying frequency-domain features for an eeg-based pain measurement system," in *2007 IEEE 33rd Annual Northeast Bioengineering Conference*. IEEE, 2007, pp. 114–115.

[3] M. Eriksson, H. Storm, A. Fremming, and J. Schollin, "Skin conductance compared to a combined behavioural and physiological pain measure in newborn infants," *Acta paediatrica*, vol. 97, no. 1, pp. 27–30, 2008.

[4] E. K. Choo, W. Magruder, C. J. Montgomery, J. Lim, R. Brant, and J. M. Ansermino, "Skin conductance fluctuations correlate poorly with postoperative self-report pain measures in school-aged children," *The Journal of the American Society of Anesthesiologists*, vol. 113, no. 1, pp. 175–182, 2010.

[5] M. L. Loggia, M. Juneau, and M. C. Bushnell, "Autonomic responses to heat pain: Heart rate, skin conductance, and their relation to verbal ratings and stimulus intensity," *PAIN®*, vol. 152, no. 3, pp. 592–598, 2011.

[6] P. Panavaranan and Y. Wongsawat, "Eeg-based pain estimation via fuzzy logic and polynomial kernel support vector machine," in *The 6th 2013 Biomedical Engineering International Conference*. IEEE, 2013, pp. 1–4.

[7] J. J. Meeuse, M. S. Löwik, S. A. Löwik, E. Aarden, A. M. van Roon, R. O. Gans, M. van Wijhe, J. D. Lefrandt, and A. K. Reyners, "Heart rate variability parameters do not correlate with pain intensity in healthy volunteers," *Pain Medicine*, vol. 14, no. 8, pp. 1192–1201, 2013.

[8] P. Werner, A. Al-Hamadi, R. Niese, S. Walter, S. Gruss, and H. C. Traue, "Towards pain monitoring: Facial expression, head pose, a new database, an automatic system and remaining challenges," in *Proceedings of the British Machine Vision Conference*, 2013, pp. 1–13.

[9] S. Walter, S. Gruss, K. Limbrecht-Ecklundt, H. C. Traue, P. Werner, A. Al-Hamadi, N. Diniz, G. M. d. Silva, and A. O. Andrade, "Automatic pain quantification using autonomic parameters," *Psychology & Neuroscience*, vol. 7, no. 3, pp. 363–380, 2014.

[10] Y. Chu, X. Zhao, J. Yao, Y. Zhao, and Z. Wu, "Physiological signals based quantitative evaluation method of the pain," *IFAC Proceedings Volumes*, vol. 47, no. 3, pp. 2981–2986, 2014.

[11] P. Werner, A. Al-Hamadi, R. Niese, S. Walter, S. Gruss, and H. C. Traue, "Automatic pain recognition from video and biomedical signals," in *2014 22nd International Conference on Pattern Recognition*. IEEE, 2014, pp. 4582–4587.

[12] M. Kächele, P. Werner, A. Al-Hamadi, G. Palm, S. Walter, and F. Schwenker, "Bio-visual fusion for person-independent recognition of pain intensity," in *International Workshop on Multiple Classifier Systems*. Springer, 2015, pp. 220–230.

[13] M. Kächele, P. Thiam, M. Amirian, P. Werner, S. Walter, F. Schwenker, and G. Palm, "Multimodal data fusion for person-independent, continuous estimation of pain intensity," in *International Conference on Engineering Applications of Neural Networks*. Springer, 2015, pp. 275–285.

[14] L. J. Hadjileontiadis, "Eeg-based tonic cold pain characterization using wavelet higher order spectral features," *IEEE Transactions on Biomedical Engineering*, vol. 62, no. 8, pp. 1981–1991, 2015.

[15] S. Gruss, R. Treister, P. Werner, H. C. Traue, S. Crawcour, A. Andrade, and S. Walter, "Pain intensity recognition rates via biopotential feature patterns with support vector machines," *PloS one*, vol. 10, no. 10, p. e0140330, 2015.

[16] M. Kächele, M. Amirian, P. Thiam, P. Werner, S. Walter, G. Palm, and F. Schwenker, "Adaptive confidence learning for the personalization of pain intensity estimation systems," *Evolving Systems*, vol. 8, no. 1, pp. 71–83, 2017.

[17] M. Kächele, P. Thiam, M. Amirian, F. Schwenker, and G. Palm, "Methods for person-centered continuous pain intensity assessment from bio-physiological channels," *IEEE Journal of Selected Topics in Signal Processing*, vol. 10, no. 5, pp. 854–864, 2016.

[18] A. J. Hautala, J. Karppinen, and T. Seppänen, "Short-term assessment of autonomic nervous system as a potential tool to quantify pain experience," in *2016 38th Annual International Conference of the IEEE Engineering in Medicine and Biology Society (EMBC)*. IEEE, 2016, pp. 2684–2687.

[19] D. Lopez-Martinez and R. Picard, "Multi-task neural networks for personalized pain recognition from physiological signals," in *2017 Seventh International Conference on Affective Computing and Intelligent Interaction Workshops and Demos (ACIIW)*. IEEE, 2017, pp. 181–184.

[20] T. Adjei, W. Von Rosenberg, V. Goverdovsky, K. Powezka, U. Jaffer, and D. P. Mandic, "Pain prediction from ecg in vascular surgery," *IEEE journal of Translational Engineering in Health and Medicine*, vol. 5, pp. 1–10, 2017.

[21] P. Thiam and F. Schwenker, "Multi-modal data fusion for pain intensity assessment and classification," in *2017 Seventh International Conference on Image Processing Theory, Tools and Applications (IPTA)*. IEEE, 2017, pp. 1–6.

[22] Y. Chu, X. Zhao, J. Han, and Y. Su, "Physiological signal-based method for measurement of pain intensity," *Frontiers in neuroscience*, vol. 11, p. 279, 2017.

[23] V. Vijayakumar, M. Case, S. Shirinpour, and B. He, "Quantifying and characterizing tonic thermal pain across subjects from eeg data using random forest models," *IEEE Transactions on Biomedical Engineering*, vol. 64, no. 12, pp. 2988–2996, 2017.

[24] M. Jiang, R. Mieronkoski, A. M. Rahmani, N. Hagelberg, S. Salanterä, and P. Liljeberg, "Ultra-short-term analysis of heart rate variability for real-time acute pain monitoring with wearable electronics," in *2017 IEEE International Conference on Bioinformatics and Biomedicine (BIBM)*. IEEE, 2017, pp. 1025–1032.

[25] B. T. Susam, M. Akcakaya, H. Nezamfar, D. Diaz, X. Xu, V. R. de Sa, K. D. Craig, J. S. Huang, and M. S. Goodwin, "Automated pain assessment using electrodermal activity data and machine learning," in *2018 40th Annual International Conference of the IEEE Engineering in Medicine and Biology Society (EMBC)*. IEEE, 2018, pp. 372–375.

[26] D. Lopez-Martinez and R. Picard, "Continuous pain intensity estimation from autonomic signals with recurrent neural networks," in *2018 40th Annual International Conference of the IEEE Engineering in Medicine and Biology Society (EMBC)*. IEEE, 2018, pp. 5624–5627.

[27] M. A. Haque, R. B. Bautista, F. Noroozi, K. Kulkarni, C. B. Laursen, R. Irani, M. Bellantonio, S. Escalera, G. Anbarjafari, K. Nasrollahi *et al.*, "Deep multimodal pain recognition: a database and comparison of spatio-temporal visual modalities," in *2018 13th IEEE International Conference on Automatic Face & Gesture Recognition (FG 2018)*. IEEE, 2018, pp. 250–257.

[28] M. Jiang, R. Mieronkoski, E. Syrjälä, A. Anzanpour, V. Terävä, A. M. Rahmani, S. Salanterä, R. Aantaa, N. Hagelberg, and P. Liljeberg, "Acute pain intensity monitoring with the classification of multiple physiological parameters," *Journal of clinical monitoring and computing*, vol. 33, no. 3, pp. 493–507, 2019.

[29] E. K. Naeini, S. Shahhosseini, A. Subramanian, T. Yin, A. M. Rahmani, and N. Dutt, "An edge-assisted and smart system for real-time pain monitoring," in *2019 IEEE/ACM International Conference on Connected Health: Applications, Systems and Engineering Technologies (CHASE)*. IEEE, 2019, pp. 47–52.

[30] E. Campbell, A. Phinyomark, and E. Scheme, "Feature extraction and selection for pain recognition using peripheral physiological signals," *Frontiers in neuroscience*, vol. 13, p. 437, 2019.

[31] P. Werner, A. Al-Hamadi, S. Gruss, and S. Walter, "Twofold-multimodal pain recognition with the x-ite pain database," in *2019 8th International Conference on Affective Computing and Intelligent Interaction Workshops and Demos (ACIIW)*. IEEE, 2019, pp. 290–296.

[32] H. Lim, B. Kim, G.-J. Noh, and S. K. Yoo, "A deep neural network-based pain classifier using a photoplethysmography signal," *Sensors*, vol. 19, no. 2, p. 384, 2019.

[33] P. Thiam, P. Bellmann, H. A. Kestler, and F. Schwenker, "Exploring deep physiological models for nociceptive pain recognition," *Sensors*, vol. 19, no. 20, p. 4503, 2019.

[34] M. T. Uddin and S. Canavan, "Multimodal multilevel fusion for sequential protective behavior detection and pain estimation," in *2020 15th IEEE International Conference on Automatic Face and Gesture Recognition (FG 2020)*. IEEE, 2020, pp. 844–848.

[35] M. S. Salekin, G. Zamzmi, J. Hausmann, D. Goldgof, R. Kasturi, M. Kneusel, T. Ashmeade, T. Ho, and Y. Sun, "Multimodal neonatal procedural and postoperative pain assessment dataset," *Data in Brief*, vol. 35, p. 106796, 2021.

[36] P. Thiam, H. Hihn, D. A. Braun, H. A. Kestler, and F. Schwenker, "Multi-modal pain intensity assessment based on physiological signals: A deep learning perspective," *Frontiers in Physiology*, vol. 12, p. 720464, 2021.

[37] P. Gouverneur, F. Li, W. M. Adamczyk, T. M. Szikszay, K. Luedtke, and M. Grzegorzek, "Comparison of feature extraction methods for physiological signals for heat-based pain recognition," *Sensors*, vol. 21, no. 14, p. 4838, 2021.
[38] A. Badura, A. Masłowska, A. Myśliwiec, and E. Piętka, "Multimodal signal analysis for pain recognition in physiotherapy using wavelet scattering transform," *Sensors*, vol. 21, no. 4, p. 1311, 2021.
[39] P. Thiam, V. Kessler, M. Amirian, P. Bellmann, G. Layher, Y. Zhang, M. Velana, S. Gruss, S. Walter, H. C. Traue *et al.*, "Multi-modal pain intensity recognition based on the senseemotion database," *IEEE Transactions on Affective Computing*, vol. 12, no. 3, pp. 743–760, 2019.
[40] E. Othman, P. Werner, F. Saxen, M.-A. Fiedler, and A. Al-Hamadi, "An automatic system for continuous pain intensity monitoring based on analyzing data from uni-, bi-, and multi-modality," *Sensors*, vol. 22, no. 13, p. 4992, 2022.
[41] P. Bellmann, P. Thiam, H. A. Kestler, and F. Schwenker, "Machine learning-based pain intensity estimation: Where pattern recognition meets chaos theory—an example based on the biovid heat pain database," *IEEE Access*, vol. 10, pp. 102 770–102 777, 2022.
[42] F. Pouromran, Y. Lin, and S. Kamarthi, "Personalized deep bi-lstm rnn based model for pain intensity classification using eda signal," *Sensors*, vol. 22, no. 21, p. 8087, 2022.
[43] B. D. Winslow, R. Kwasinski, K. Whirlow, E. Mills, J. Hullfish, and M. Carroll, "Automatic detection of pain using machine learning," *Frontiers in Pain Research*, vol. 3, p. 1044518, 2022.
[44] S. Moscato, S. Orlandi, A. Giannelli, R. Ostan, and L. Chiari, "Automatic pain assessment on cancer patients using physiological signals recorded in real-world contexts," in *2022 44th Annual International Conference of the IEEE Engineering in Medicine & Biology Society (EMBC)*. IEEE, 2022, pp. 1931–1934.
[45] Y. Lin, Y. Xiao, L. Wang, Y. Guo, W. Zhu, B. Dalip, S. Kamarthi, K. L. Schreiber, R. R. Edwards, and R. D. Urman, "Experimental exploration of objective human pain assessment using multimodal sensing signals," *Frontiers in Neuroscience*, vol. 16, p. 831627, 2022.
[46] E. F. Teel, D. D. Ocay, S. Blain-Moraes, and C. E. Ferland, "Accurate classification of pain experiences using wearable electroencephalography in adolescents with and without chronic musculoskeletal pain," *Frontiers in Pain Research*, vol. 3, p. 991793, 2022.
[47] P. Gouverneur, F. Li, K. Shirahama, L. Luebke, W. M. Adamczyk, T. M. Szikszay, K. Luedtke, and M. Grzegorzek, "Explainable artificial intelligence (xai) in pain research: Understanding the role of electrodermal activity for automated pain recognition," *Sensors*, vol. 23, no. 4, p. 1959, 2023.
[48] J. O. Pinzon-Arenas, Y. Kong, K. H. Chon, and H. F. Posada-Quintero, "Design and evaluation of deep learning models for continuous acute pain detection based on phasic electrodermal activity," *IEEE Journal of Biomedical and Health Informatics*, 2023.
[49] L. Chen, Z. Zhang, R. Han, L. Du, Z. Li, S. Liu, D. Huang, and H. Zhou, "Painvision-based evaluation of brain potentials: a novel approach for quantitative pain assessment," *Frontiers in Bioengineering and Biotechnology*, vol. 11, p. 1197070, 2023.
[50] M. Jiang, R. Rosio, S. Salanterä, A. M. Rahmani, P. Liljeberg, D. S. da Silva, V. H. C. de Albuquerque, and W. Wu, "Personalized and adaptive neural networks for pain detection from multi-modal physiological features," *Expert Systems with Applications*, vol. 235, p. 121082, 2024.
